# Supplementary material for: Mitochondrial phylogenomics reveals deep relationships of scarab beetles (Coleoptera, Scarabaeidae)
Source: PLoS One. 2022 Dec 13;17(12):e0278820. doi: 10.1371/journal.pone.0278820 (PMC9746968; doi:10.1371/journal.pone.0278820)
Supplement: Attachment — Submitted filename: PONE-D-22-21573_R1 new.pdf [file pone.0278820.s015.pdf]

PLOS ONE

Mitochondrial phylogenomics reveals deep relationships of scarab beetles (Coleoptera, Scarabaeidae)

--Manuscript Draft--

**Manuscript Number:**	PONE-D-22-21573R1
**Article Type:**	Research Article
**Full Title:**	Mitochondrial phylogenomics reveals deep relationships of scarab beetles (Coleoptera, Scarabaeidae)
**Short Title:**	Mitochondrial phylogenomics of scarab beetles
**Corresponding Author:**	Nan Song, Ph.D.<br>College of plant protection<br>Zhengzhou, CHINA
**Keywords:**	Cetoniinae, Melolonthinae; mitochondrial genome; Phylogeny
**Abstract:**	In this study, we newly sequenced the complete mitochondrial genomes (mitogenomes) of two phytophagous scarab beetles, and investigated the deep level relationships within Scarabaeidae combined with other published beetle mitogenome sequences. The complete mitogenomes of Dicronocephalus adamsi Pascoe (Cetoniinae) and Amphimallon sp. (Melolonthinae) are 15,563 bp and 17,433 bp in size, respectively. Both mitogenomes have the typical set of 37 genes (13 protein-coding genes, 22 transfer RNA genes, two ribosomal RNA genes) and an A+T-rich region, with the same gene arrangement found in the majority of beetles. The secondary structures for ribosomal RNA genes (rrnL and rrnS) were inferred by comparative analysis method. Results from phylogenetic analyses provide support for major lineages and current classification of Scarabaeidae. Amino acid data recovered Scarabaeidae as monophyletic. The Scarabaeidae was split into two clades. One clade contained the subfamilies Scarabaeinae and Aphodiinae. The other major clade contained the subfamilies Dynastinae, Rutelinae, Cetoniinae, Melolonthinae and Sericini. The monophyly of Scarabaeinae, Aphodiinae, Dynastinae, Cetoniinae and Sericini were strongly supported. The Scarabaeinae was the sister group of Aphodiinae. The Cetoniinae was sister to the Dynastinae + Rutelinae clade. The Melolonthinae was a non-monophyletic group. The removal of fast-evolving sites from nucleotide dataset using a pattern sorting method (OV-sorting) supported the family Scarabaeidae as a monophyletic group. At the tribe level, the Onthophagini was non-monophyletic with respect to Oniticellini. Ateuchini was sister to a large clade comprising the tribes Onthophagini, Oniticellini and Onitini. Eurysternini was a sister group of the Phanaeini + Ateuchini clade.
**Order of Authors:**	Shibao Guo
	Xingyu Lin
	Nan Song, Ph.D.
**Response to Reviewers:**	Responses to comments<br><br>Dear Reviewers,<br><br>Thank you for your very careful review of our paper, and for the comments, corrections and suggestions that ensued. A major revision of the paper has been carried out to take all of them into account. And in the process, we believe the paper has been significantly improved.<br><br>In the present "Responses to comments", we sequentially address all of the points raised in each of the comments made by different reviewers.<br><br><br>Reviewer #1:<br>Q1: Breeschoten et al. 2016 for which much of the included sequences were published

in,
Response: This reference has been cited in Table S1.
In the old version, we find the references associated with the mitogenome sequences from GenBank. Although much of the included sequences are from the Breeschoten et al. 2016, the references in GenBank are not updated where the state of “JOURNAL” is still “Unpublished”. According to your comment, we read the paper of Breeschoten et al. 2016 and checked the mitogenome sequences included in it. Indeed, much of the included sequences come from this paper.
Thank you for this comment.

Q2: phylogenies that address the monophyly of the Scarabaeidae including McKenna et al. 2014 and Gunter et al. 2016 especially the most recent papers with dense gene or taxon sampling- are relatively well resolved and provide a broader snap-shot of the deep level relationship that this paper attempts to address.
Response: After careful inspection, we found that McKenna et al. 2014 you mentioned might be the one of McKenna et al. 2015 (please see details in the following).
McKenna DD, Farrell BD, Caterino MS, Farnum CW, Hawks DC, Maddison DR, et al. Phylogeny and evolution of Staphyliniformia and Scarabaeiformia: forest litter as a stepping-stone for diversification of non-phytophagous beetles. Syst. Entomol. 2015; 40: 35–60. doi: 10.1111/syen.12093
This paper included the content of addressing the monophyly of the Scarabaeidae. This reference has been cited in this version. Page 12 Line 303, and Page 15 Lines 395-396.
In addition, we also read carefully the paper of Gunter et al. 2016 and cited it in this version. Page 3 Lines 52, 55 and Page 14 Lines 356-360.

Q3: It is extremely surprising to me that the Lucanidae rendered Scarabaeidae para-phyletic in some analysis. It is generally considered one of the earliest branching members of the superfamily as supported by molecular result and the deep fossil record. This surprising result may indicate that the mt-genome phylogeny is either insufficiently sampled at a taxon-level or is inappropriate for resolving relationships. When analyzed by amino acids and removing the 3rd codon results were more congruent with past analyses.
Response: I am sorry that this is a mistake in writing. In the preliminary analysis on the datasets with the different taxon sampling, some resulting trees might please Lucanidae nested within the Scarabaeidae. In the present version, there are no trees with Lucanidae nested within the Scarabaeidae. The associated sentence has been deleted.

Q4: Ultimately, while I think there are elements of this paper that are novel- the vast majority of this is not. The novelty in this paper revolves around the new genomes, including their descriptions of structure, unfortunately the particular species sequenced do not add novel insights in to poorly resolved relationships in Scarabaeidae. Large components of this paper are just a reanalysis of previously published data from other papers. The results and discussion to not add any new insights in to relationships within the family or subfamilies. For example, the entire section on tribal relationships and nesting behaviors is unnecessary as it is a synthesis of other work that has been discussed in detail in other, sometimes uncited, papers.
Response: The paper of Breeshoten et al. (2016) has been cited in this version (Page 15 Lines 390-391, and Page 16 Lines 397-399). We added some discussions on the comparisons between our results and theirs. With regard to the comments from another reviewer, we added the tribe labels in Figures 4-5 and retained this section in the text.

Q5: Furthermore, I disagree with the statement on line 364 that it’s sufficiently sampled (beyond the ingroup of Breeshoten’s work) to address internal relationships, as a number of tribes are not includes, are represented by a single taxon or multiple species in the same genus. I suspect the authors are not familiar with much of the scarab literature because other unanswered questions / unconfirmed relationships in the Scarabaeidae, such as the putative paraphyly of Rutelinae, were not discussed.
Response: In this version, we compared the taxon sampling of Rutelinae with the two prior studies of Ahrens 2014 and Gunter 2016. For the monophyly of Rutelinae, we have added new discussions in Page 14 Lines 350-366.

Powered by Editorial Manager® and ProduXion Manager® from Aries Systems Corporation

	Q6: I would have liked to see a summary of taxon sampling within the text. The figures were neat and informative. Please note that some taxonomic names within the phylogeny are out of date, for example Digonthophaagus bonasus.<br>Response: The species name Onthophagus bonasus is from NCBI database (KU739459) https://www.ncbi.nlm.nih.gov/nuccore/KU739459.1/.<br>In this version, we have corrected Onthophagus bonasus to Digitonthophagus bonasus throughout the trees.<br><br><br>Reviewer #2:<br>Q1: 1). Coprophagy. Aphodinae and a significant minority of Scarabaeinae aren't strict dung feeders. They are generalist saprovores, consuming dead/rotting plant material of which dung is a specialised dietary type (this is reflected in the fact that few scarabaeine dung beetle consume dung from non-herbivorous vertebrates). These dietary distinctions and their evolution are discussed at length in Gunter et al. 2016 PLoS One 11 e0153570. It is also worth noting that these distinctions exclusively concern adult diet - almost all Scarabs are saprovores as larvae, specialisation between phyto, sapro and coprophagy is only found in adults.<br>Response: Thanks for this comment. The corresponding statements have been revised and the paper of Gunter et al. 2016 has been cited in this version. Please see details in Page 3 Lines 42-44, 52-55.<br><br>Q2: 2) Literature comparisons. The taxon sampling used here, while extensive from the mt genome point of view, is restricted relative to the available molecular phylogenies of the family. For example, just 3sp of Aphedinae, 6 Rutelinae (and just 3 genera) etc. The comparisons to prior literature thus need to be more refined that direct equivalence of subfamily or tribes between the present study and past ones.<br>It is necessary to examine if the representatives within each higher taxon are comparable studies, or if the ones included here represent only particular clades included in other studies.<br>For example Rutelinae is paraphyletic in both Gunter 2016 and Ahrens 2014, forming 3 clades and Dynastinae within the Rutelinae. The relationships found in the present study could be completely accurate if only species from 1 of those three clades were included i.e. both subfamilies monophletic, it would just be misleading as Rutelinae is undersampled to test those prior relationships. Similar issues with the Scarabaeinae.<br>Response: In this version, we compared the taxon sampling of Rutelinae with the two prior studies of Ahrens 2014 and Gunter 2016. For the monophyly of Rutelinae, we have added new discussions in Page 14 Lines 350-366.<br><br>Q3: I would like to see a more detailed examination of how well sampled clades are rather than just statements as to monophyly and relationship with other clades. Additionally, as devote substantial discussion space to tribal relationships in Scarabaeinae, tribe labels should be labelled on your figure for ease of reading. This section could also benefit from more extensive use of recent literature on behavioural evolution as noted above for Coprophagy.<br>Response: We compared the taxon sampling of the major clade within Scarabaeidae included in this study to the prior studies, particularly the studies of Breeshoten et al. (2016), Ahrens et al. (2014) and Gunter et al. (2016). We added the discussions on the tribe relationships within Scarabaeinae (Page 15 Lines 390-391, and Page 16 Lines 397-399) and the monophyly of Rutelinae (Page 14 Lines 350-366). In addition, we added the tribe labels in Figures 4-5.
**Additional Information:**	
**Question**	**Response**
**Financial Disclosure**<br><br>Enter a financial disclosure statement that describes the sources of funding for the work included in this submission. Review the submission guidelines for detailed requirements. View published research articles from *PLOS ONE* for specific	This research was funded by the National Natural Science Foundation of China, grant number U1904104, and the Foundation of Central Laboratory of Xinyang Agriculture and Forestry Uni-versity, grant number FCL202003.

Powered by Editorial Manager® and ProduXion Manager® from Aries Systems Corporation

examples.<br><br>This statement is required for submission and **will appear in the published article** if the submission is accepted. Please make sure it is accurate.<br><br>**Unfunded studies**<br>Enter: *The author(s) received no specific funding for this work.*<br><br>**Funded studies**<br>Enter a statement with the following details:<br>• Initials of the authors who received each award<br>• Grant numbers awarded to each author<br>• The full name of each funder<br>• URL of each funder website<br>• Did the sponsors or funders play any role in the study design, data collection and analysis, decision to publish, or preparation of the manuscript?<br>• **NO** - Include this sentence at the end of your statement: *The funders had no role in study design, data collection and analysis, decision to publish, or preparation of the manuscript.*<br>• **YES** - Specify the role(s) played.<br><br>* typeset	
**Competing Interests**<br><br>Use the instructions below to enter a competing interest statement for this submission. On behalf of all authors, disclose any competing interests that could be perceived to bias this work—acknowledging all financial support and any other relevant financial or non-financial competing interests.<br><br>This statement is required for submission and **will appear in the published article** if the submission is accepted. Please make sure it is accurate and that any funding sources listed in your Funding Information later in the submission form are also declared in your Financial Disclosure statement.	The authors declare no conflict of interest.

View published research articles from *PLOS ONE* for specific examples.<br><br>**NO authors have competing interests**<br><br>Enter: *The authors have declared that no competing interests exist.*<br><br>**Authors with competing interests**<br><br>Enter competing interest details beginning with this statement:<br><br>*I have read the journal's policy and the authors of this manuscript have the following competing interests: [insert competing interests here]*<br><br>* typeset	
**Ethics Statement**<br><br>Enter an ethics statement for this submission. This statement is required if the study involved:<br><br>• Human participants<br>• Human specimens or tissue<br>• Vertebrate animals or cephalopods<br>• Vertebrate embryos or tissues<br>• Field research<br><br>Write "N/A" if the submission does not require an ethics statement.<br><br>General guidance is provided below. Consult the submission guidelines for detailed instructions. **Make sure that all information entered here is included in the Methods section of the manuscript.**	No specific permits were required for the insect specimens collected for this study. These specimens were collected on the field of Zhengzhou. The field studies did not involve endangered or protected species. The two insect species sequenced are all common beetle species in China and are not included in the ‘‘List of Protected Animals in China”.

Powered by Editorial Manager® and ProduXion Manager® from Aries Systems Corporation

**Format for specific study types**<br><br>**Human Subject Research (involving human participants and/or tissue)**<br>• Give the name of the institutional review board or ethics committee that approved the study<br>• Include the approval number and/or a statement indicating approval of this research<br>• Indicate the form of consent obtained (written/oral) or the reason that consent was not obtained (e.g. the data were analyzed anonymously)<br><br>**Animal Research (involving vertebrate animals, embryos or tissues)**<br>• Provide the name of the Institutional Animal Care and Use Committee (IACUC) or other relevant ethics board that reviewed the study protocol, and indicate whether they approved this research or granted a formal waiver of ethical approval<br>• Include an approval number if one was obtained<br>• If the study involved *non-human primates*, add *additional details* about animal welfare and steps taken to ameliorate suffering<br>• If anesthesia, euthanasia, or any kind of animal sacrifice is part of the study, include briefly which substances and/or methods were applied<br><br>**Field Research**<br><br>Include the following details if this study involves the collection of plant, animal, or other materials from a natural setting:<br>• Field permit number<br>• Name of the institution or relevant body that granted permission	
**Data Availability**<br><br>Authors are required to make all data underlying the findings described fully available, without restriction, and from the time of publication. PLOS allows rare exceptions to address legal and ethical concerns. See the PLOS Data Policy and FAQ for detailed information.	Yes - all data are fully available without restriction

A Data Availability Statement describing where the data can be found is required at submission. Your answers to this question constitute the Data Availability Statement and **will be published in the article**, if accepted.<br><br>**Important:** Stating 'data available on request from the author' is not sufficient. If your data are only available upon request, select 'No' for the first question and explain your exceptional situation in the text box.<br><br>Do the authors confirm that all data underlying the findings described in their manuscript are fully available without restriction?	
**Describe where the data may be found in full sentences. If you are copying our sample text, replace any instances of XXX with the appropriate details.**<br><br>• If the data are **held or will be held in a public repository**, include URLs, accession numbers or DOIs. If this information will only be available after acceptance, indicate this by ticking the box below. For example: *All XXX files are available from the XXX database (accession number(s) XXX, XXX.).*<br>• If the data are all contained **within the manuscript and/or Supporting Information files**, enter the following: *All relevant data are within the manuscript and its Supporting Information files.*<br>• If neither of these applies but you are able to provide **details of access elsewhere**, with or without limitations, please do so. For example:<br><br>*Data cannot be shared publicly because of [XXX]. Data are available from the XXX Institutional Data Access / Ethics Committee (contact via XXX) for researchers who meet the criteria for access to confidential data.*<br><br>*The data underlying the results presented in the study are available from (include the name of the third party*	The mitogenome sequences of Dicronocephalus adamsi and Amphimallon sp. have been deposited at GenBank, with the accession number of MZ955033 and ON529251.

and contact information or URL).<br>• This text is appropriate if the data are owned by a third party and authors do not have permission to share the data.<br><br>* typeset	
Additional data availability information:	

Manuscript Click here to access/download;Manuscript;Main_text.docx

Mitochondrial phylogenomics reveals deep relationships of scarab beetles (Coleoptera, Scarabaeidae)

Shibao Guo[1*], Xingyu Lin[2], Nan Song[2*]

1 Xinyang Agriculture and Forestry University, Xinyang, Henan, China

2 College of Plant Protection, Henan Agricultural University, Zhengzhou, Henan, China

* Corresponding Authors:

Shibao Guo, Nan Song

E-mail: sbguo510@163.com; songnan@henau.edu.cn

Abstract

In this study, we newly sequenced the complete mitochondrial genomes (mitogenomes) of two phytophagous scarab beetles, and investigated the deep level relationships within Scarabaeidae combined with other published beetle mitogenome sequences. The complete mitogenomes of *Dicronocephalus adamsi* Pascoe (Cetoniinae) and *Amphimallon* sp. (Melolonthinae) are 15,563 bp and 17,433 bp in size, respectively. Both mitogenomes have the typical set of 37 genes (13 protein-coding genes, 22 transfer RNA genes, two ribosomal RNA genes) and an A+T-rich region, with the same gene arrangement found in the majority of beetles. The secondary structures for ribosomal RNA genes (*rrnL* and *rrnS*) were inferred by comparative analysis method. Results from phylogenetic analyses provide support for major lineages and current classification of Scarabaeidae. Amino acid data recovered Scarabaeidae as monophyletic. The Scarabaeidae was split into two clades. One clade contained the subfamilies Scarabaeinae and Aphodiinae. The other major clade contained the subfamilies Dynastinae, Rutelinae, Cetoniinae, Melolonthinae and Sericini. The monophyly of Scarabaeinae, Aphodiinae, Dynastinae, Cetoniinae and Sericini were strongly supported. The Scarabaeinae was the sister group of Aphodiinae. The Cetoniinae was sister to the Dynastinae + Rutelinae clade. The Melolonthinae was a non-monophyletic group. The removal of fast-evolving sites from nucleotide dataset using a pattern sorting method (OV-sorting) supported the family Scarabaeidae as a monophyletic group. At the tribe level, the Onthophagini was non-monophyletic with respect to Oniticellini. Ateuchini was sister to a large clade comprising the tribes Onthophagini, Oniticellini and Onitini. Eurysternini was a sister group of the Phanaeini + Ateuchini clade.

Keywords: Cetoniinae, Melolonthinae; mitochondrial genome; phylogeny

Introduction

The family Scarabaeidae is one of the largest and most diverse groups of beetles, with more than 30,000 described species worldwide [1-5]. According to feeding preferences, this family was divided into two major groups, namely the saprophagous and phytophagous lineages. Most of the saprophagous scarabs are specialist dung feeders in adults, which are often called dung beetles and well known to most people due to their relatively large body size, significantly ecological importance and the sacred symbols of *Scarabaeus* in ancient Egypt. Dung beetles are one of the primary utilizers of mammalian dung on Earth. Many species of phytophagous scarab beetles are economically damaging pests because of the high levels of herbivory. For example, the Japanese beetle *Popillia japonica* is broadly invasive and causes millions of dollars of damage worldwide [6-8].

Despite their enormous ecological and economic importance, the phylogenetic relationships within Scarabaeidae remain controversial. The saprophagous scarabs comprise two major subfamily groups: Scarabaeinae and Aphodiinae [9]. The former are predominantly specialist dung feeders, while the latter are generalists feeding on dead or decaying matter like leaf litter and rotting fruits [1, 9]. In addition, some species classified in Hybosoridae, Ochodaeidae, Geotrupidae and other Scarabaeoidea groups were once considered to be the close relatives of Scarabaeinae and Aphodiinae, and all of them constituted the Laparosticti [10]. The phytophagous scarab beetles are traditionally grouped into four main subfamilies (Melolonthinae, Cetoniinae, Dynastinae, and Rutelinae) [11-13]. The phytophagous scarabs are called as Pleurosticti [14]. The Laparosticti and Pleurosticti are often characterized by location of the spiracles [15, 16], but see [17]. The division of Scarabaeoidea into Laparosticti and Pleurosticti is currently considered as untenable. Besides the six main subfamilies mentioned above, a few other much smaller subfamilies were recognized in the Scarabaeidae, such as Aclopinae, Allidiostomatinae, Euchirinae, Orphninae and Pachypodinae [12, 18].

The monophyly of the family Scarabaeidae as a whole and the relationships among

the subfamilies have been contentious. Grebennikov and Scholtz (2004) [18] used larval morphological characters to infer the higher-level relationships of Scarabaeoidea. Their results did not support the monophyly of Scarabaeidae. The subfamilies Scarabaeinae and Aphodiinae was recovered as separate clades [18]. In contrast, the molecular phylogenetic study using the 28S rDNA and 18S rDNA sequence data found strong support for the family Scarabaeidae [11]. The monophyly of the dung beetle clade (Scarabaeinae + Aphodiinae) was also supported [11]. A phylogenetic analysis of the Coleoptera using 95 protein-coding genes also supported the monophyly of Scarabaeidae [19]. The phylogenetic analyses based on the mitochondrial genome (mitogenome) sequences consistently recovered a monophyletic Scarabaeidae [3, 20], though with the limited sampling of Scarabaeoidea. However, a phylogenetic analysis of Scarabaeoidea based on four DNA markers (the 18S rDNA and 28S rDNA genes, and the mitochondrial *cox1* and *rrnL* genes) recovered a non-monophyletic Scarabaeidae [21]. Besides the question of Scarabaeidae monophyly, the subfamilies Aphodiinae and Melolonthinae were also questioned in the previous studies [3, 11]. In fact, the classification for the Scarabaeidae differs between systematists and regions. For example, Ahrens often recognized Sericini to be a tribe of Melolonthinae (e.g., [13, 22, 23]). The tribe Sericini as a member of Melolonthinae was also supported by the study based on the 18S and 28S rDNA sequence data [12]. However, Ahrens et al (2014) recovered Melolonthinae to be a paraphyletic assemblage, with Sericini being a separate clade [21]. Several recent phylogenetic analyses based on mitogenomes often recovered sericine chafers outside Melolonthinae, and tended to support the subfamily status of Sericinae in Scarabaeidae [3, 5, 24].

The main aims of the present study are threefold: (i) to test the monophyly of Scarabaeidae; (ii) to elucidate the phylogenetic relationships among the subfamilies within Scarabaeidae; (iii) to explore the utility of the mitogenome sequence data for these phylogenetic relationships. To perform this study, we sequenced two new mitogenomes from the Cetoniinae (*Dicronocephalus adamsi* Pascoe, 1863) and the Melolonthinae (*Amphimallon* sp.). Combined with the published mitogenomes of

Scarabaeoidea construct the most comprehensive datasets of the scarab mitogenome sequences to date. DNA sequence data were analyzed under various inference methods and different data recoding schemes.

Material and methods

Ethics statement

No specific permits were required for the insect specimens collected for this study. These specimens were collected on the field of Zhengzhou. The field studies did not involve endangered or protected species. The two insect species sequenced are all common beetle species in China and are not included in the ‘‘List of Protected Animals in China”.

Taxonomic sampling

To evaluate the monophyly of Scarabaeidae and the phylogenetic relationships within this family, we chose a set of ingroup and outgroup species satisfying the following conditions: (i) inclusion of members of several major subfamilies, which ensured covering the major clades of the dung scarabs and the phytophagous scarabs; (ii) inclusion of major families within the superfamily Scarabaeoidea; (iii) choosing the Hydrophilidae to root the tree. A total of 118 ingroup species were included: 70 species from Scarabaeinae, three from Aphodiinae, ten from Dynastinae, six from Rutelinae, 12 from Cetoniinae, 14 from Melolonthinae, and three from Sericini (or Sericinae) (Table S1). Outgroup taxa for Scarabaeidae belonged to the closely-related families Trogidae (two species), Glaresidae (one species), Geotrupidae (two species), Lucanidae (four species), Hybosoridae (three species), and Passalidae (three species). The more distantly related Hydrophilidae (14 species) were also used to be the non-Scarabaeoidea outgroups. In this study, the classification within Scarabaeoidea followed those of Smith (2006) [25].

Three mitogenome sequences downloaded from the public database GenBank

(Scarabaeidae sp. BMNH 1274750, Scarabaeidae sp. BMNH 1274752, Scarabaeidae sp. BMNH 1274753). Three sequences were originally classified at the family level. We re-conducted molecular identification using the mitochondrial *cox1* gene. In addition, combined with the resulting trees inferred in the present study, we assigned Scarabaeidae sp. BMNH 1274752 to the tribe Canthonini (Scarabaeinae) and both Scarabaeidae sp. BMNH 1274750 and Scarabaeidae sp. BMNH 1274753 to the tribe Ateuchini (Scarabaeinae).

Sequencing of complete mitogenomes

Specimen collection and DNA extraction

The adult specimens of *D. adamsi* and *Amphimallon* sp. were collected at Zhengzhou, Henan province, China, in July 2019 and 2020, respectively. The specimens were preserved in absolute ethanol and stored at -80 ℃ until DNA extraction. Total genomic DNA was extracted from leg muscle tissue using the TIANamp Genomic DNA Kit (TIANGEN BIOTECH CO., LTD), following the manufacturer's protocol.

Next generation sequencing

The extracts were delivered to BGI-Shenzhen, China for library preparation and sequencing. Libraries for sequencing were prepared from cDNA, which was sheared to produce 300-4000 bp double stranded fragments. Sequencing was performed by using the BGISEQ-500 platform, with the strategy of 100 paired-end. Low quality sequencing reads were trimmed by using NGS QC Toolkit [26]. The remaining clean data (15,947,554 reads for *D. adamsi* and 21,808,396 reads for *Amphimallon* sp.) were then assembled into contigs.

Mitogenomes assembly, annotation and analysis

Clean reads were used for *de novo* assembly of mitogneomes of using GetOrganelle

v1.7.5.2 [27]. We used the GetOrganelle animal database (-F animal_mt) to identify, filter and assemble target-associated reads.

The mitogenome contigs were initially annotated in the MITOS webserver [28], under the Metazoan RefSeq 63 set and default settings. The gene boundaries of protein-coding genes were refined by alignment with closely related scarab species. Transfer RNA (tRNA) genes were determined and the corresponding secondary structures were inferred in MITOS web [28]. Secondary structures for *rrnL* and *rrnS* genes were predicted by the comparative method, with reference to the scarab beetle *Popillia mutans* [3]. The new mitogenome sequences of *D. adamsi* and *Amphimallon* sp. have been deposited at GenBank, with the accession number of MZ955033 and ON529251.

Nucleotide compositions of the mitogenome sequences were calculated using MEGA X [29]. The strand compositional bias was examined by using AT and GC-skew values calculated by the formula: AT-skew = (A-T)/(A+T) and GC-skew = (G-C)/(G+C) [30].

Multiple sequence alignment

Sequence alignments of protein-coding genes were accomplished using TranslatorX [31], with the MAFFT algorithm [32]. The poorly aligned regions and gaps were removed by trimAl v1.4 [33], with the "-automated1 heuristic". Ribosomal and transfer RNA genes were aligned individually in MAFFT version 7 [32], with the iterative refinement option of E-INS-i. The resulting alignments were also trimmed using trimAl v1.4 [33], with the above-mentioned parameter settings. Alignments were concatenated with FASconCAT-G_v1.04 [34] to compile the following three types of datasets: 1) PCG_nt, the concatenated nucleotide sequences including 13 protein-coding genes; 2) PCG_aa, the concatenated amino acid sequences including 13 protein-coding genes; 3) PCGRNA, the concatenated nucleotide sequences including 13 protein-coding genes, two rRNA genes and 22 tRNA genes; 4) PCG_nt12, the concatenated nucleotide sequences of 13 protein-coding genes with the third codon positions removed; and 5) PCG12RNA, the concatenated nucleotide sequences of PCG_nt12, two rRNA genes

and 22 tRNA genes.

Phylogenetic analyses

Tree searches

We employed both Maximum likelihood (ML) and Bayesian inference (BI) methods to conduct phylogenetic tree reconstructions. Model selection was conducted in ModelFinder [35] for every concatenated matrix used herein, with the alignment partitioned by gene.

ML trees were reconstructed from each of the matrices with IQ-TREE 1.6.12 [36] using the best-scoring substitution model for each gene partition (Table S2) as selected with ModelFinder [35]. Support for trees was assessed with 10,000 ultrafast bootstrap (BS) replicates [37].

Bayesian tree searches were performed with PhyloBayes-MPI 1.8 [38, 39]. For each dataset, we conducted two independent runs. The CAT-GTR model was used in the analyses of nucleotide alignments, while the CAT-mtZOA model was used in the analysis of amino acid alignment. Each run involved two chains, with a total length of 10,000 cycles. Convergence between chains was evaluated by examining the difference in frequency for all their bipartitions (maxdiff < 0.3). The consensus tree was built from the two runs with the *bpcomp* program discarding the first 1,000 trees as burn-in. Branch support was assessed by the clade posterior probabilities (PP).

We tested how slow and fast evolving sites could affect phylogenetic discordance. To do so, trees were generated from data matrices produced using the OV-sorting method [40]. This method ranks the originally concatenated alignment from the most variable sites to the most conserved sites based on the measurement of "observed variability" (OV) of each alignment position. Previous studies have suggested that accuracy of phylogenetic inference can be improved by the removal of the most variable sites from datasets using the OV-sorting method [41-43]. A series of shortened alignments were created by sequentially removing fast-evolving sites in 250 increments

from the 13,858 sites of the PCGRNA dataset. The OV-sorted datasets were subjected to unpartitioned ML analyses, with the parameter settings as described above.

Hypothesis Testing

The specific phylogenetic hypothesis, namely the relationships of major clades of Scarabaeidae, was further assessed by means of the four-cluster likelihood-mapping (FcLM) approach implemented in IQ-TREE 1.6.12 [36]. For the three datasets (PCG_nt, PCG_aa and PCGRNA), we used the same partition schemes and the corresponding models selected during the IQ-TREE tree search.

Results

Sequencing of mitochondrial genomes

Genome sequencing produced 15,947,554 and 21,808,396 paired-end reads for *D. adamsi* and *Amphimallon* sp., respectively. The approximately 3.53% and 0.13% of the reads resembled mitochondrial sequences. The 15,563 bp and 17,433 bp contigs were assembled for the mitogenomes of *D. adamsi* and *Amphimallon* sp.. Mean coverage of the two contigs reached 4339-fold and 266-fold.

The mitogneomes of *D. adamsi* and *Amphimallon* sp. have the typical set of 37 mitochondrial genes (13 protein-coding genes, 22 tRNA genes, and two rRNA genes) and a major noncoding region (also called control region or A+T-rich region). Both mitogenomes have the similar gene content and genome organization found in the majority of beetles. Nucleotide composition of the mitogenomes was A+T biased, 74.4% for *D. adamsi* and 73.5% for *Amphimallon* sp., respectively. For the majority strand in two scarab species, the AT-skew is positive (higher A than T content, 0.025 for *D. adamsi* and 0.056 for *Amphimallon* sp.) while the GC-skew is negative (higher C than G content, -0.27 for *D. adamsi* and -0.25 for *Amphimallon* sp.).

The complete set of 22 tRNA genes typical of insect mitogenomes [44] is present

in the two mitogenomes. The length of tRNA genes range from 62 bp to 71 bp. All tRNA gene sequences can be folded into the cloverleaf structure, except for *trnS1* in both species and *trnW* in *D. adamsi* (Figure 1 and Figure S1). The *trnS1* in both species and the *trnW* in *D. adamsi* have an unpaired stretch of 12 nucleotides or 7 nucleotides instead of the DHU arm.

The lengths of *rrnS* genes are 780 bp and 785 bp for *D. adamsi* and *Amphimallon* sp., and the lengths of *rrnL* genes are 1,303 bp and 1,329 bp, respectively. The secondary structures inferred for the *rrnL* and *rrnS* genes were similar to the secondary structure models proposed for other scarab beetles (e.g., *P. mutans*, [3]). Both *rrnL* genes' secondary structures include five major domains (I, II, IV, V and VI). The sequence spanning domain II and domain IV is very short that leads to a loss of the domain III (Figure 2 and Figure S 2). This pattern is often found in insect *rrnL* gene sequences. Five domains are composed of 41-42 helices. *rrnS* genes have three domains (I, II and III) composed of 26 helices (Figure 3 and Figure S3).

Phylogenetic inferences

The same alignment produced the same topology regardless of inference method used. In the ML and BI trees from the amino acid dataset PCG_aa (Figure 4-5), the family Scarabaeidae was well supported and split into two major clades. One clade contained the subfamilies Scarabaeinae and Aphodiinae, another included the subfamilies Dynastinae, Rutelinae, Cetoniinae, Melolonthinae and the tribe Sericini. The monophyly of Scarabaeinae, Aphodiinae, Dynastinae, Cetoniinae and Sericini were strongly supported (BS = 100, PP = 1). The node for Rutelinae received the statistically significant support (PP = 0.97) in the BI analysis, but not in the ML analysis (BS = 56). The Melolonthinae was non-monophyletic in both ML and BI trees. In the analyses based on the amino acid dataset, several sister-group relationships were consistently recovered with strong support. The Scarabaeinae was the sister group of Aphodiinae (BS = 100, PP = 1). The Cetoniinae was sister group to the Dynastinae + Rutelinae clade.

The analyses from the nucleotide datasets (PCG_nt and PCGRNA, Figure S4-S7) yielded tree topologies with the largely identical subfamily relationships in Scarabaeidae. The Scarabaeidae was subdivided into two major clades, which had the same composition and internal arrangement of the subfamilies to those of the analyses from the amino acid dataset. However, the family Scarabaeidae was non-monophyletic with respect to Hybosoridae. The subfamily Rutelinae was recovered as a monophyletic group in the nucleotide analyses. The Melolonthinae was paraphyletic, with the grouping of Cheirotonus + Holotrichia as a separate clade.

Removal of third codon positions did not change tree topological structure in the nucleotide analyses. In the ML tree from the PCG12RNA dataset (Figure S8), the outgroups (Lucanidae + (Passalidae + Hybosoridae)) was sister to the phytophagous scarab clade, which resulted in a non-monophyletic Scarabaeidae. A similar branching pattern was recovered in the ML analysis of the PCG_nt12 dataset (Figure S9). In the BI tree from the PCG12RNA dataset (Figure S10), a sister group relationship between the dung scarab clade (Scarabaeinae + Aphodiinae) and Hybosoridae (PP = 0.8) led to the paraphyletic Scarabaeidae. For the PhyloBayes analysis of the dataset PCG_nt12 (Figure S11), ambiguity represented by polytomies was found in the relationships among the dung scarab clade, the phytophagous scarab clade and the two outgroup families of Passalidae and Hybosoridae. This pattern showed that removing third codon positions resulted in the loss of phylogenetic signal for resolving the deep relationships among the groups. Despite the non-monophyly of Scarabaeidae, terminal relationships among subfamilies of the family were generally stable across analyses of nucleotide datasets.

Sequentially removing the fast-evolving sites from the PCGRNA dataset produced a series of shortened alignments, which contained increasing proportion of conserved sites. As illustrated in Figure 6, the non-phylogenetic Scarabaeidae was retrieved while the 2,000 most variable sites were included in the ML analysis. After these sites were removed, the monophyletic Scarabaeidae was recovered until 5,000 sites were removed. The nodal support value for Scarabaeidae gradually increased from the analysis of the

subset containing 11,608 positions to the analysis of the subset containing 9,608 positions. In the ML trees from the subsets further removing the fast-evolving sites in the subsequent three steps, relationships within Scarabaeidae collapsed due to information loss. After this point (subset containing 8,858 positions), the Scarabaeidae was recovered to be non-monophyletic again and the entire relationships on the tree were unresolved.

The FcLM results (Figure 7), showed support for the sister-group relationship between the Scarabaeinae + Aphodiinae clade and the clade including all other scarab subfamilies (58.5% - 79.4%), which is congruent with the ML topology inferred from the amino acid dataset. Alternative relationships had poor support: the Scarabaeinae + Aphodiinae clade being sister group to Hybosoridae (14.5% - 31.8%), and the clade including Dynastinae, Rutelinae, Cetoniinae, Melolonthinae and Sericini being sister group to Hybosoridae (2.6% - 5.7%). The FcML results suggested that the monophyletic Scarabaeidae was indeed better supported by Maximum likelihood.

Discussion

The monophyly of Scarabaeidae

Traditionally, based on morphological evidence, the scarab dung beetles and the pleurostict chafers are considered to be sister groups, both of which constitute the monophyletic Scarabaeidae [45-48]. The Scarabaeidae was also recovered in recent molecular studies [19, 20, 49]. However, the Scarabaeidae was not supported by other analyses based on morphological characters [18, 50], multi-locus sequence data [21, 51-53] and single-copy nuclear protein-coding gene data [54]. In the present study, the monophyly of Scarabaeidae was strongly confirmed by ML analysis (BS = 88) based on amino acid data. In the BI analysis based on the same dataset, the Scarabaeidae was recovered, but with only weak support (PP = 0.86).

Analysis based on the nucleotide subsets produced by the OV-sorting methods showed that removal of the most variable sites has a significant effect on the

phylogenetic inference of relationships of Scarabaeidae. The subsets including more fast-evolving sites supported Scarabaeidae to be a non-monophyletic group. Whereas, the subsets containing a relatively large number of conserved positions can recover a monophyletic Scarabaeidae.

Overall, we found that all strongly supported nodes in Scarabaeidae were congruent between topologies from the amino acid dataset and the OV-sorted alignments containing more conserved positions. However, the amino acid dataset and the nucleotide dataset with full positions produced conflict result: the former recovered a monophyletic Scarabaeidae, but the latter retrieved a non-monophyletic Scarabaeidae. The possible explanation for the topological difference between phylograms may be base compositional heterogeneity, substitution model misspecification and the potential tree-building artifacts (e.g., long-branch attraction). The FcLM analyses suggested that a sister-group relationship between the dung scarab clade and the phytophagous scarab clade is better supported by ML.

The interrelationships among subfamilies within Scarabaeidae

Despite the distinct branching pattern on the monophyly of Scarabaeidae inferred from the amino acid dataset and the nucleotide dataset with full positions, both types of data provided the supported phylogenetic hypotheses in splitting Scarabaeidae into two major clades and the interrelationships between subfamilies in each of the clades.

With regard to the dung beetle clade, all analyses recovered the reciprocal monophyly of Scarabaeinae and Aphodiinae. Moreover, the sister group relationship of Scarabaeinae and Aphodiinae was strongly supported (BS = 100, PP = 1). This result was highly concordant with previous morphological [46, 50] and molecular studies [11, 20, 51, 53], but contrasted with the studies of Grebennikov and Scholtz (2004) [6] and Hunt et al. (2007) [52].

The phytophagous scarab clade has long been recognized as a monophyletic group. The earlier studies based on the morphological characters provided strong support for the clade [46, 48, 55]. This clade was further supported by the subsequent analyses

based on molecular data [11, 20, 21, 51]. However, the relationships among the subfamilies within the clade remained unstable. In our analyses based on the mitogenomic data, the monophyly of the phytophagous scarab clade was strongly supported (BS = 100, PP = 1). Moreover, we recovered a well-resolved topology with several strongly supported subfamily groups within the clade. The Sericini were unequivocally recovered as sister to the remaining pleurosticts. A sister group relationship between Dynastinae and Rutelinae was confirmed. This arrangement was in accordance with the previous morphological [46, 48, 56] and molecular analyses [11, 24]. Both Dynastinae and Rutelinae formed the sister group of Cetoniinae. This branching order was consistent with that in the study of Ahrens et al. (2014)[21].

In this study, only six mitogenome sequences were included for the Rutelinae due to the data availability of the subfamily. These sequences are from four genera classified in two tribes: the *Adoretus* of Adoretini, and the *Mimela*, *Phyllopertha* and *Popillia* of Anomalini. All analyses clustered the six Rutelinae species in a clade, with Adoretini as a sister group of Anomalini. However, in some analyses (ML PCG_aa, ML PCG_nt, BI PCG_nt, BI PCGRNA, and BI PCG_nt12), there was weak support for a Adoretini + Anomalini sister-group relationship ($BS < 70$, $PP < 0.9$). In two prior studies of [9, 21], Rutelinae were recovered as a paraphyletic group. Based on the analyses of four DNA markers (*18S*, *28S*, *16S*, and *cox1*), Ahrens et al. (2014) [21] recovered Adoretini as a sister group of a clade composed of Pachydemini and Dynastinae. Gunter et al. (2016) [9] used the different molecular data (*28S*, *16S*, *12S*, and *cox1*) to yield a similar result to Ahrens et al. (2014) [21]. Rutelinae was recovered as a paraphyletic grade, with Adoretini being sister to Dynastinae [21]. The Anoplognathini and the Rutelini + Anomalini clade formed the independent lineages [21], respectively. Compared with the previous studies [9, 21], our taxon sampling for Rutelinae was incomplete. Increased taxon sampling is expected to generate a more conclusive estimate on the monophyly of Rutelinae.

The Melolonthinae was recovered to be a paraphyletic group. In fact, the monophyly of Melolonthinae has been questioned in previous studies. Ahrens et al.

(2014) [21] recovered Melolonthinae as a paraphyletic group based on the analysis of multi-locus data. A prior mitogenomic analysis also supported Melolonthinae as a paraphyletic assemblage [3].

Tribal relationships within Scarabaeinae and implications for nesting behaviors

Dung beetles in Scarabaeinae were once classified into two main groups based on their rolling and tunneling behavior, namely Scarabaeinae and Coprinae [57]. The former group included six tribes Eurysternini, Deltochilini, Eucraniini, Gymnopleurini, Scarabaeini and Sisyphini, and the latter contained six tribes Coprini, Oniticellini, Onitini, Onthophaghini, Ateuchini and Phanaeini. Although this system was widely accepted by the subsequent systematists on the Scarabaeinae, some questions have been raised on the classification of the subfamily. Montreuil (1998) [58] assigned the genera of Dichotomiini to the tribe Coprini and renamed the tribe Dichotomiini as Ateuchini. Scholtz et al (2009) [59] suggested that Dichotomiini may be a polyphyletic group. A molecular phylogenetic analysis recovered the rolling Canthonini and tunnelling Dichotomiini to be polyphyletic [60]. The phylogenetic analysis based on the morphological characters further supported the polyphyly of Dichotomiini [61]. A morphological study by Philips et al (2004) [62] did not support the monophyly of the Ateuchini. Bouchard (2011) [63] revised the system of Balthasar (1963) [57] to include 11 tribes in Scarabaeinae, of which Eurysternini was considered as a subtribe in Oniticellini.

For the tribal relationships within Scarabaeinae, our results are consistent with the study of Breeschoten et al. (2016) [64]. The Onthophagini with the extensive taxon sampling was a non-monophyletic group. The monophyletic Oniticellini was nested within a large clade composed of all members of Onthophagini. A previous morphological study has proposed to treat Oniticellini as a subgroup of Onthophagini [65]. The molecular phylogenetic analyses always placed Oniticellini within

Onthophagini [48, 49]. This study based on the mitogenomic data confirmed the pattern recovered in the previous analyses [48, 49, 64, 65]. In the ML analysis of amino acid data, the Onitini was recovered as a sister group of the clade including Onthophagini and Oniticellini. A similar result was obtained in the phylogeny of previous studies [64-68].

Ateuchini represented by the species of *Xinidium* sp. was sister to a large clade comprising the tribes Onthophagini, Oniticellini and Onitini. The placement of Ateuchini is similar to that in the study of multi-locus sequence data [67]. The remaining species from Ateuchini were distributed in three clades at the base of the tree, which rendered a non-monophyletic Ateuchini. Based on the analysis of adult morphological characters, Montreuil (1998) [58] recognized Ateuchini to be non-monophyletic.

The Phanaeini represented by a single species of *Coprophanaeus* sp. BMNH679884 was placed in an intermediate position between *Dichotomius* and *Canthidium*. This placement of Phanaeini was incongruent with the previous molecular phylogeny inferred by Monaghan et al. (2007) [67]. In the tree of Monaghan et al. (2007) [68], the *Dichotomius* and *Canthidium* were sister groups, both of which were sister to the Phanaeini + Eucraniini clade. Due to the limited availability of mitogenome sequences, no taxon sampling of Eucraniini was included in the present study. Therefore, there is need for further sequence mitogenomes from the Eucraniini to elucidate the relationships among the tribes.

The monophyletic Eurysternini was a sister group of the clade including Phanaeini and Ateuchini. The *Sarophorus* + *Copris* clade was sister to all other Scarabaeinae lineages. The early diverging position of *Sarophorus* was congruent with Mlambo et al. (2015) [67]. However, the sister group relationship between *Sarophorus* and *Copris* was different from that in Mlambo et al. (2015) [66]. In the tree from Mlambo et al. (2015) [66], both genera had a distant relationship.

According to literatures [62, 66], the *Sarophorus* are non-dung-feeding while Coprini are tunneling. The Eurysternini and Canthonini are recognized as rollers. The

tribes Dichotomiini, Phanaeini, Onitini, Oniticellini and Onthophagini and the genus *Xinidium* are often considered as tunnellers. An early phylogenetic analysis using morphological characters did not support the hypothesis of the roller/tunneller classification in Scarabaeinae [69]. In the phylogenetic framework estimated by the current mitogenome sequences, rolling and tunneling behaviors were intermixed throughout the Scarabaeinae lineages. This pattern was consistent with the prior studies [62, 66, 67].

Conclusion

In this study, we newly sequenced two mitogenomes from scarab beetles belonging to Cetoniinae and Melolonthinae. Combined with the published beetle mitogenomes, we constructed the most comprehensive mitogenome sequence datasets to date. In which, we have included all major subfamilies of Scarabaeidae. Results provide support for major lineages and current classification of Scarabaeidae. The amino acid dataset and the OV-sorted alignments containing more conserved positions supported the family Scarabaeidae as a monophyletic group. This result was congruent with some morphological studies [45-48], but contrasted with several molecular studies [21, 51-54]. The sister group relationships between Scarabaeinae and Aphodiinae and between Dynastinae and Rutelinae were confirmed. The Melolonthinae was non-monophyletic. The present mitogenome data supported Sericini as an independent clade that indicated a subfamily rank of this group. At the tribe level, the Onthophagini was non-monophyletic with respect to Oniticellini.

Supporting Information files:

Figure S1: The secondary structures of tRNA genes inferred for *Amphimallon* sp..

Figure S2: The secondary structure of *rrnL* inferred for *Amphimallon* sp..

Figure S3: The secondary structure of *rrnS* inferred for *Amphimallon* sp..

Figure S4: Phylogenetic tree of relationships within Scarabaeidae based on Maximum

likelihood analysis of the dataset PCG_nt.

Figure S5: Phylogenetic tree inferred from the dataset PCG_nt based on Bayesian inference.

Figure S6: Phylogenetic tree of relationships within Scarabaeidae based on Maximum likelihood analysis of the dataset PCGRNA.

Figure S7: Phylogenetic tree inferred from the dataset PCGRNA based on Bayesian inference.

Figure S8: Phylogenetic tree of relationships within Scarabaeidae based on Maximum likelihood analysis of the dataset PCG12RNA.

Figure S9: Phylogenetic tree of relationships within Scarabaeidae based on Maximum likelihood analysis of the dataset PCG_nt12.

Figure S10: Phylogenetic tree inferred from the dataset PCG12RNA based on Bayesian inference.

Figure S11: Phylogenetic tree inferred from the dataset PCG_nt12 based on Bayesian inference.

Table S1: Taxa included in this study.

Table S2: The partitioning schemes and best-fitting modes selected by ModelFinder for the dataset (A) PCG_nt, (B) PCG_aa, (C) PCGRNA, (D) PCG_nt12 and (E) PCG12RNA.

Author Contributions: Conceptualization, S.G. and N.S.; methodology, S.G., N.S. and X.L.; software, N.S. and X.L.; validation, S.G., N.S. and X.L.; formal analysis, N.S. and X.L.; investigation, N.S. and X.L.; resources, S.G. and N.S.; data curation, N.S. and X.L.; writing—original draft preparation, N.S.; writing—review and editing, S.G. and N.S.; visualization, S.G. and N.S.; supervision, S.G.; project administration, S.G. and N.S.; funding acquisition, S.G. and N.S. All authors have read and agreed to the published version of the manuscript.

Data Availability Statement: The mitogenome sequences of *Dicronocephalus adamsi*

and *Amphimallon* sp. have been deposited at GenBank, with the accession number of MZ955033 and ON529251.

Conflicts of Interest: The authors declare no conflict of interest.

References

1. Scholtz C, Grebennikov V. In Handbook of Zoology Vol. IV, Arthropoda, Part II, Insecta. Kristensen NP, Beutel RG. Eds. Coleoptera Vol 1, Chap 12. Walter De Gruyter, Berlin; 2005.
2. Huang S, Sheng P, Zhang H. Isolation and identification of cellulolytic bacteria from the gut of Holotrichia parallela larvae (Coleoptera: Scarabaeidae). Int J Mol Sci. 2012;13(3):2563-77.
3. Song N, Zhang H. The mitochondrial genomes of phytophagous scarab beetles and systematic implications. J Insect Sci. 2018;18(6):11.
4. Jeong JS, Kim MJ, Kim I. The mitochondrial genome of the dung beetle, Copris tripartitus, with mitogenomic comparisons within Scarabaeidae (Coleoptera). Int J Biol Macromol. 2020;144:874-91.
5. Ayivi SPG, Tong Y, Storey KB, Yu D-N, Zhang J-Y. The Mitochondrial Genomes of 18 New Pleurosticti (Coleoptera: Scarabaeidae) Exhibit a Novel trnQ-NCR-trnI-trnM Gene Rearrangement and Clarify Phylogenetic Relationships of Subfamilies within Scarabaeidae. Insects. 2021;12(11):1025.
6. Hengeveld R. Dynamics of biological invasions: Springer Science & Business Media; 1989.
7. Jameson ML, Paucar-Cabrera A, Solís A. Synopsis of the New World genera of Anomalini

(Coleoptera: Scarabaeidae: Rutelinae) and description of a new genus from Costa Rica and Nicaragua. Ann Entomol Soc Am. 2003;96(4):415-32.

8. Mori N, Santoiemma G, Glazer I, Gilioli G, Ciampitti M, Cavagna B, et al. Management of Popillia japonica in container-grown nursery stock in Italy. Phytoparasitica. 2022;50(1):83-9.

9. Gunter NL, Weir TA, Slipinksi A, Bocak L, Cameron SL. If dung beetles (Scarabaeidae: Scarabaeinae) arose in association with dinosaurs, did they also suffer a mass co-extinction at the K-Pg boundary? PloS ONE. 2016;11(5):e0153570.

10. Woodruff R. The Scarab Beetles of Florida. Pt. I. The Laparosticti. Arthropods of Florida and Neighbouring Land Areas. 1973;8:1-220.

11. Eberle J, Myburgh R, Ahrens D. The evolution of morphospace in phytophagous scarab chafers: No competition-no divergence? PloS ONE. 2014;9(5):e98536.

12. Smith AB, Hawks DC, Heraty JM. An overview of the classification and evolution of the major scarab beetle clades (Coleoptera: Scarabaeoidea) based on preliminary molecular analyses. Coleopts Bull. 2006;60(5):35-46.

13. Ahrens D, Vogler AP. Towards the phylogeny of chafers (Sericini): analysis of alignment-variable sequences and the evolution of segment numbers in the antennal club. Mol Phylogenet Evol. 2008;47(2):783-98.

14. W E. Naturgeschichte der Insecten Deutschlands. Erste Abteilung, Coleoptera. Berlin: Nicolaische Buchhandlung. 1847;9:35-46.

15. Jameson ML, Ratcliffe BC. Series Scarabaeiformia Crowson 1960, Superfamily Scarabaeoidea Latreille 1802. Papers in Entomology. 2002:50.

16. Anlaş S, Keith D, Tezcan S. Notes on the seasonal dynamics of some coprophagous

Scarabaeoidea (Coleoptera) species in Manisa province, western Anatolia. Turk entomol derg. 2011;35(3):447-60.

17. Ritcher PO. Spiracles of adult Scarabaeoidea (Coleoptera) and their phylogenetic significance. I. The abdominal spiracles. Ann Entomol Soc Am. 1969;62(4):869-80.

18. Grebennikov VV, Scholtz CH. The basal phylogeny of Scarabaeoidea (Insecta: Coleoptera) inferred from larval morphology. Invertebr Syst. 2004;18(3):321-48.

19. Zhang S-Q, Che L-H, Li Y, Liang D, Pang H, Ślipiński A, et al. Evolutionary history of Coleoptera revealed by extensive sampling of genes and species. Nat Commun. 2018;9(1):1-11.

20. Timmermans MJ, Barton C, Haran J, Ahrens D, Culverwell CL, Ollikainen A, et al. Family-Level Sampling of Mitochondrial Genomes in Coleoptera: Compositional Heterogeneity and Phylogenetics. Genome Biol Evol. 2015;8(1):161-75. Epub 20151208. doi: 10.1093/gbe/evv241. PubMed PMID: 26645679; PubMed Central PMCID: PMCPMC4758238.

21. Ahrens D, Schwarzer J, Vogler AP. The evolution of scarab beetles tracks the sequential rise of angiosperms and mammals. P Roy Soc B-Biol Sci. 2014;281(1791):20141470.

22. Ahrens D. The phylogeny of Sericini and their position within the Scarabaeidae based on morphological characters (Coleoptera: Scarabaeidae). Syst Entomol. 2006;31(1):113-44.

23. Ahrens D. Taxonomic changes and an updated catalogue for the Palaearctic Sericini (Coleoptera: Scarabaeidae: Melolonthinae). Zootaxa. 2007;1504(1):1–51-1–.

24. Li Y, Nie R, Lu Y, Lee S, Zhao Z, Wu L, et al. Seven new mitochondrial genomes of phytophagous scarab beetles (Coleoptera: Scarabaeidae) and phylogenetic implications. Zootaxa. 2022;5138(3):324-38.

25. Smith AB. A review of the family-group names for the superfamily Scarabaeoidea (Coleoptera) with corrections to nomenclature and a current classification. Coleopts Bull. 2006;60(mo5):144-204.

26. Patel RK, Jain M. NGS QC Toolkit: a toolkit for quality control of next generation sequencing data. PloS ONE. 2012;7(2):e30619.

27. Jin J-J, Yu W-B, Yang J-B, Song Y, DePamphilis CW, Yi T-S, et al. GetOrganelle: a fast and versatile toolkit for accurate de novo assembly of organelle genomes. Genome Biol. 2020;21(1):1-31.

28. Bernt M, Bleidorn C, Braband A, Dambach J, Donath A, Fritzsch G, et al. A comprehensive analysis of bilaterian mitochondrial genomes and phylogeny. Mol Phylogenet Evol. 2013;69(2):352-64.

29. Kumar S, Stecher G, Li M, Knyaz C, Tamura K. MEGA X: molecular evolutionary genetics analysis across computing platforms. Mol Biol Evol. 2018;35(6):1547.

30. Perna NT, Kocher TD. Patterns of nucleotide composition at fourfold degenerate sites of animal mitochondrial genomes. J Mol Evol. 1995;41(3):353-8.

31. Abascal F, Zardoya R, Telford MJ. TranslatorX: multiple alignment of nucleotide sequences guided by amino acid translations. Nucleic Acids Res. 2010;38(suppl_2):W7-W13.

32. Katoh K, Standley DM. MAFFT multiple sequence alignment software version 7: improvements in performance and usability. Mol Biol Evol. 2013;30(4):772-80.

33. Capella-Gutiérrez S, Silla-Martínez JM, Gabaldón T. trimAl: a tool for automated alignment trimming in large-scale phylogenetic analyses. Bioinf. 2009;25(15):1972-3.

34. Kück P, Longo GC. FASconCAT-G: extensive functions for multiple sequence alignment

preparations concerning phylogenetic studies. Front Zool. 2014;11(1):1-8.

35. Kalyaanamoorthy S, Minh BQ, Wong TK, Von Haeseler A, Jermiin LS. ModelFinder: fast model selection for accurate phylogenetic estimates. Nat Methods. 2017;14(6):587-9.

36. Nguyen L-T, Schmidt HA, Von Haeseler A, Minh BQ. IQ-TREE: a fast and effective stochastic algorithm for estimating maximum-likelihood phylogenies. Mol Biol Evol. 2015;32(1):268-74.

37. Minh BQ, Nguyen MAT, von Haeseler A. Ultrafast approximation for phylogenetic bootstrap. Mol Biol Evol. 2013;30(5):1188-95.

38. Lartillot N, Philippe H. A Bayesian mixture model for across-site heterogeneities in the amino-acid replacement process. Mol Biol Evol. 2004;21(6):1095-109.

39. Lartillot N, Rodrigue N, Stubbs D, Richer J. PhyloBayes MPI: phylogenetic reconstruction with infinite mixtures of profiles in a parallel environment. Syst Biol. 2013;62(4):611-5.

40. Goremykin VV, Nikiforova SV, Bininda-Emonds OR. Automated removal of noisy data in phylogenomic analyses. J Mol Evol. 2010;71(5):319-31.

41. Goremykin VV, Nikiforova SV, Biggs PJ, Zhong B, Delange P, Martin W, et al. The evolutionary root of flowering plants. Syst Biol. 2013;62(1):50-61.

42. Parks M, Cronn R, Liston A. Separating the wheat from the chaff: mitigating the effects of noise in a plastome phylogenomic data set from Pinus L.(Pinaceae). BMC Evol Biol. 2012;12(1):1-17.

43. Zhong B, Deusch O, Goremykin VV, Penny D, Biggs PJ, Atherton RA, et al. Systematic error in seed plant phylogenomics. Genome Biol Evol. 2011;3:1340-8.

44. Cameron S. Insect mitochondrial genomics: implications for evolution and phylogeny.

Annu Rev Entomol. 2014;59(1):95-117.

45. Browne D, Scholtz C. Phylogeny of the families of Scarabaeoidea (Coleoptera) based on characters of the hindwing articulation, hindwing base and wing venation. Syst Entomol. 1995;20(3):145-73.

46. Browne J, Scholtz CH. Evolution of the scarab hindwing articulation and wing base: a contribution toward the phylogeny of the Scarabaeidae (Scarabaeoidea: Coleoptera). Syst Entomol. 1998;23(4):307-26.

47. Browne J, Scholtz CH. A phylogeny of the families of Scarabaeoidea (Coleoptera). Syst Entomol. 1999;24(1):51-84.

48. Howden H. Larval and adult characters of Frickius Germain, its relationship to the Geotrupini, and a phytogeny of some major taxa in the Scarabaeoidea (Insecta: Coleoptera). Can J Zool. 1982;60(11):2713-24.

49. Mckenna DD, Farrell BD, Caterino MS, Farnum CW, Hawks DC, Maddison DR, et al. Phylogeny and evolution of Staphyliniformia and Scarabaeiformia: forest litter as a stepping stone for diversification of nonphytophagous beetles. Syst Entomol. 2015;40(1):35-60.

50. Lawrence JF, Ślipiski A, Seago AE, Thayer MK, Newton AF, Marvaldi AE, editors. Phylogeny of the Coleoptera based on morphological characters of adults and larvae. Annales zoologici; 2011: Museum and Institute of Zoology, Polish Academy of Sciences.

51. Bocak L, Barton C, Crampton-Platt A, Chesters D, Ahrens D, Vogler AP. Building the C oleoptera tree-of-life for> 8000 species: composition of public DNA data and fit with L innaean classification. Syst Entomol. 2014;39(1):97-110.

52. Hunt T, Bergsten J, Levkanicova Z, Papadopoulou A, John OS, Wild R, et al. A

comprehensive phylogeny of beetles reveals the evolutionary origins of a superradiation. Science. 2007;318(5858):1913-6.

53. Mckenna DD, Wild AL, Kanda K, Bellamy CL, Beutel RG, Caterino MS, et al. The beetle tree of life reveals that C oleoptera survived end-P ermian mass extinction to diversify during the C retaceous terrestrial revolution. Syst Entomol. 2015;40(4):835-80.

54. Cai C, Tihelka E, Giacomelli M, Lawrence JF, Ślipiński A, Kundrata R, et al. Integrated phylogenomics and fossil data illuminate the evolution of beetles. Royal Society Open Science. 2022;9(3):211771.

55. Browne D, Scholtz CH. The morphology of the hind wing articulation and wing base of the Scarabaeoidea (Coleoptera) with some phylogenetic implications: Zoologisches Forschungsinstitut und Museum Alexander Koenig; 1996.

56. Jameson ME. Phylogenetic analysis of the subtribe Rutelina and revision of the Rutela generic groups (Coleoptera: Scarabaeidae: Rutelinae: Rutelini): University of Kansas; 1997.

57. V B. Scarabaeidae und Aphodiidae der Palaearktischen und Orientalischen Region (Coleoptera, Lamellicornia) Verlag Tschechosl. Akad Wissenschaft, Prague Berlin: Nicolaische Buchhandlung. 1963.

58. Montreuil O, editor Analyse phylogénétique et paraphylie des Coprini et Dichotomiini (Coleoptera: Scarabaeidae). Scénario biogéographique. Annales de la Société entomologique de France; 1998.

59. Scholtz C DA, Kryger U. Section C. . Phylogeny of the Scarabaeinae. . Evolutionary Biology and Conservation of Dung Beetles. 2009;225-237.

60. Sole CL, Scholtz CH. Did dung beetles arise in Africa? A phylogenetic hypothesis based

on five gene regions. Mol Phylogenet Evol. 2010;56(2):631-41.

61. Tarasov S, Genier F. Innovative Bayesian and parsimony phylogeny of dung beetles (Coleoptera, Scarabaeidae, Scarabaeinae) enhanced by ontology-based partitioning of morphological characters. PloS ONE. 2015;10(3):e0116671.

62. Philips TK, Pretorius E, Scholtz CH. A phylogenetic analysis of dung beetles (Scarabaeinae: Scarabaeidae): unrolling an evolutionary history. Invertebr Syst. 2004;18(1):53-88.

63. Bouchard P, Bousquet Y, Davies AE, Alonso-Zarazaga MA, Lawrence JF, Lyal CH, et al. Family-group names in Coleoptera (Insecta). ZooKeys. 2011;(88):1.

64. Breeschoten T, Doorenweerd C, Tarasov S, Vogler AP. Phylogenetics and biogeography of the dung beetle genus Onthophagus inferred from mitochondrial genomes. Mol Phylogenet Evol. 2016;105:86-95.

65. Tarasov SI, Solodovnikov AY. Phylogenetic analyses reveal reliable morphological markers to classify mega-diversity in Onthophagini dung beetles (Coleoptera: Scarabaeidae: Scarabaeinae). Cladistics. 2011;27(5):490-528.

66. Tarasov S, Dimitrov D. Multigene phylogenetic analysis redefines dung beetles relationships and classification (Coleoptera: Scarabaeidae: Scarabaeinae). BMC Evol Biol. 2016;16(1):1-19.

67. Mlambo S, Sole CL, Scholtz CH. A molecular phylogeny of the African Scarabaeinae (Coleoptera: Scarabaeidae). 2015.

68. Monaghan MT, Inward DJ, Hunt T, Vogler AP. A molecular phylogenetic analysis of the Scarabaeinae (dung beetles). Mol Phylogenet Evol. 2007;45(2):674-92.

69. Zunino M. Essai préliminaire sur l'évolution des armures génitales des Scarabaeinae, par rapport à la taxonomie du groupe et à l'évolution du comportement de nidification [Col. Scarabaeidae]. Bulletin de la Societe entomologique de France. 1983;88(7):531-42.

Figure legends

Figure 1. The secondary structures of tRNA genes inferred for *Dicronocephalus adamsi*. Dashes in-dicate Watson-Crick base pairing. Dots indicate G-U base pairs.

Figure 2. The secondary structure of rrnL inferred for *Dicronocephalus adamsi*. Dashes indicate Watson-Crick base pairing. Dots indicate G-U base pairs. Red roman numbers denote the domains, and blue numbers denote the helices.

Figure 3. The secondary structure of rrnS inferred for *Dicronocephalus adamsi*. Dashes indicate Watson-Crick base pairing. Dots indicate G-U base pairs. Red roman numbers denote the domains, and blue numbers denote the helices.

Figure 4. Phylogenetic tree inferred from the dataset PCG_aa using IQ-TREE under the models selected by ModelFinder. In the phylogenetic tree, node numbers show the bootstrap support values. Scale bar represents substitutions/site. Bold denotes the newly sequenced species. Colors identify the subfamily groups.

Figure 5. Phylogenetic tree inferred from the dataset PCG_aa using PhyloBayes-MPI under the model CAT-mtZOA. In the phylogenetic tree, numbers around nodes indicate posterior proba-bilities. Scale bar represents substitutions/site. Bold denotes the newly sequenced species. Colors identify the subfamily groups.

Figure 6. Phylogenetic tree inferred in IQ-TREE using the subset with 2000 sites removed from the dataset PCGRNA. Node numbers show the bootstrap support values. Scale bar represents sub-stitutions/site. Colors identify the subfamily groups.

Figure 7. Results of the Four-cluster likelihood mapping (FcLM) analyses. (A) Alternative hy-potheses on the relationships of major clades of Scarabaeidae. (B) FcLM

results for the concatenated amino acid (PCG_aa) and nucleotide (PCG_nt, PCGRNA, PCG_nt12 and PCG12RNA) alignments.

Figure 1. The secondary structures of tRNA genes inferred for Dicronocephalus adamsi.

Click here to access/download;Figure;Figure 1.tif

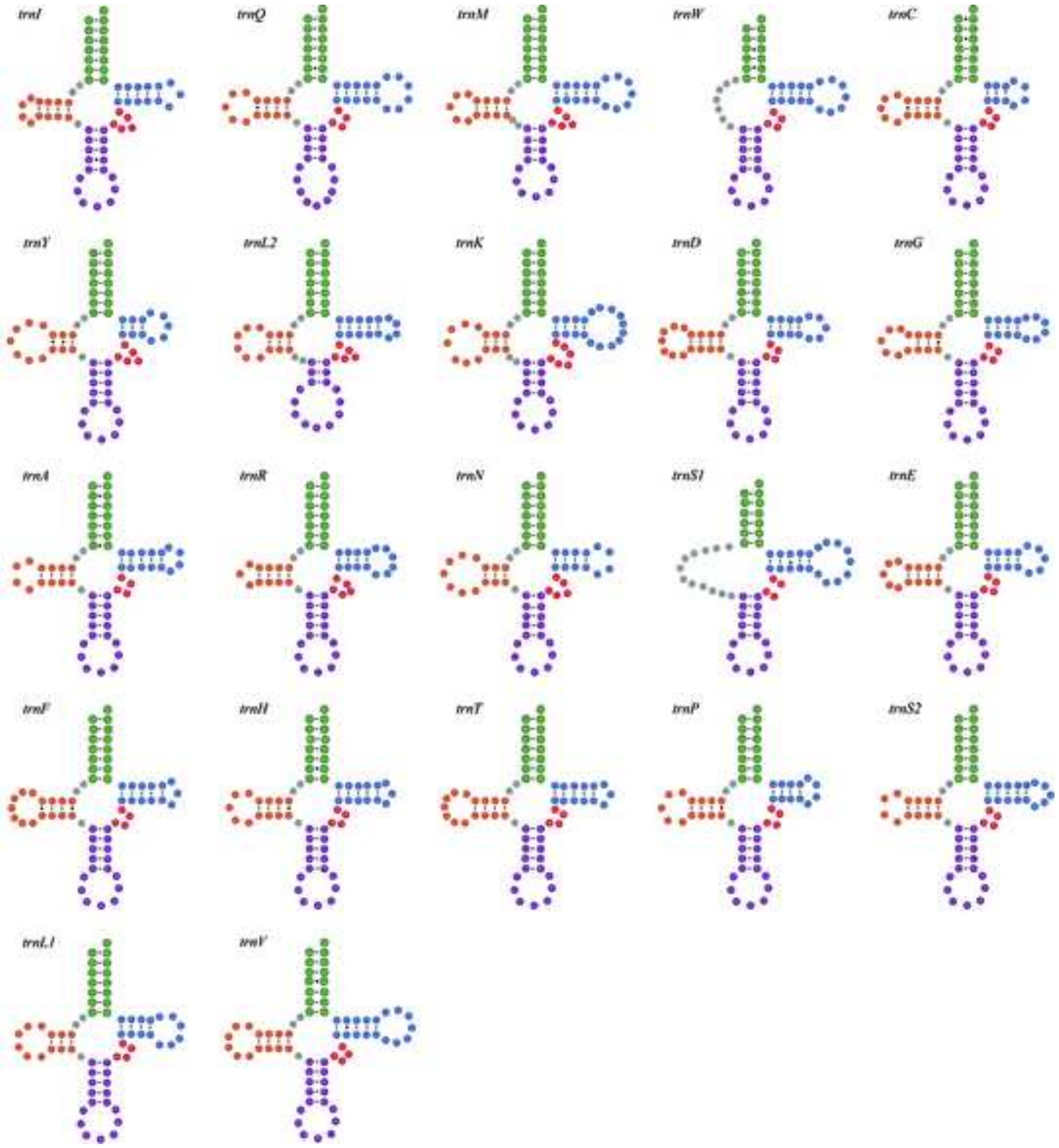

Figure 2. The secondary structure of rrnL inferred for Dicronocephalus adamsi.

Click here to access/download;Figure;Figure 2.tif

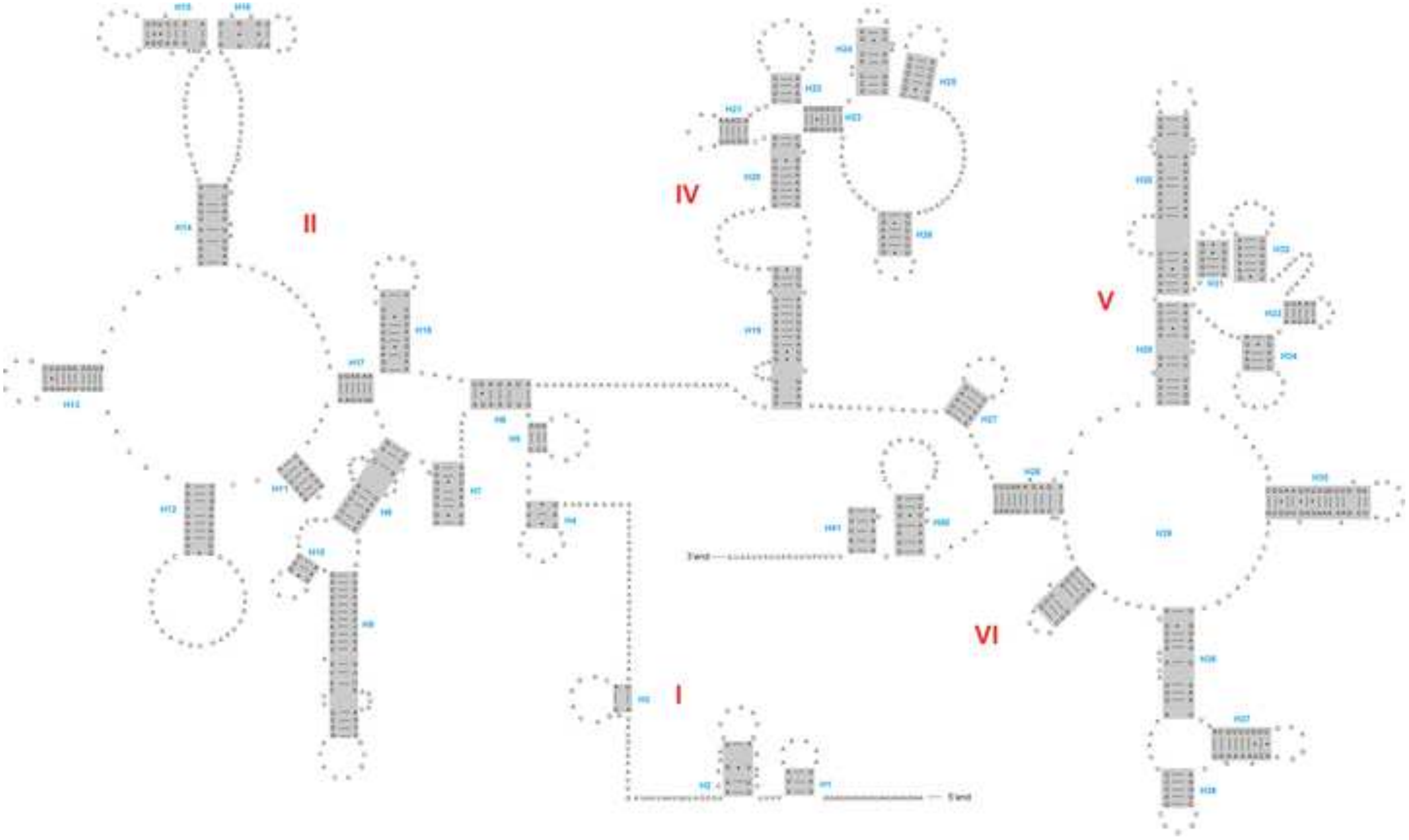

Figure 3. The secondary structure of rrnS inferred for Dicronocephalus adamsi.

Click here to access/download;Figure;Figure 3.tif

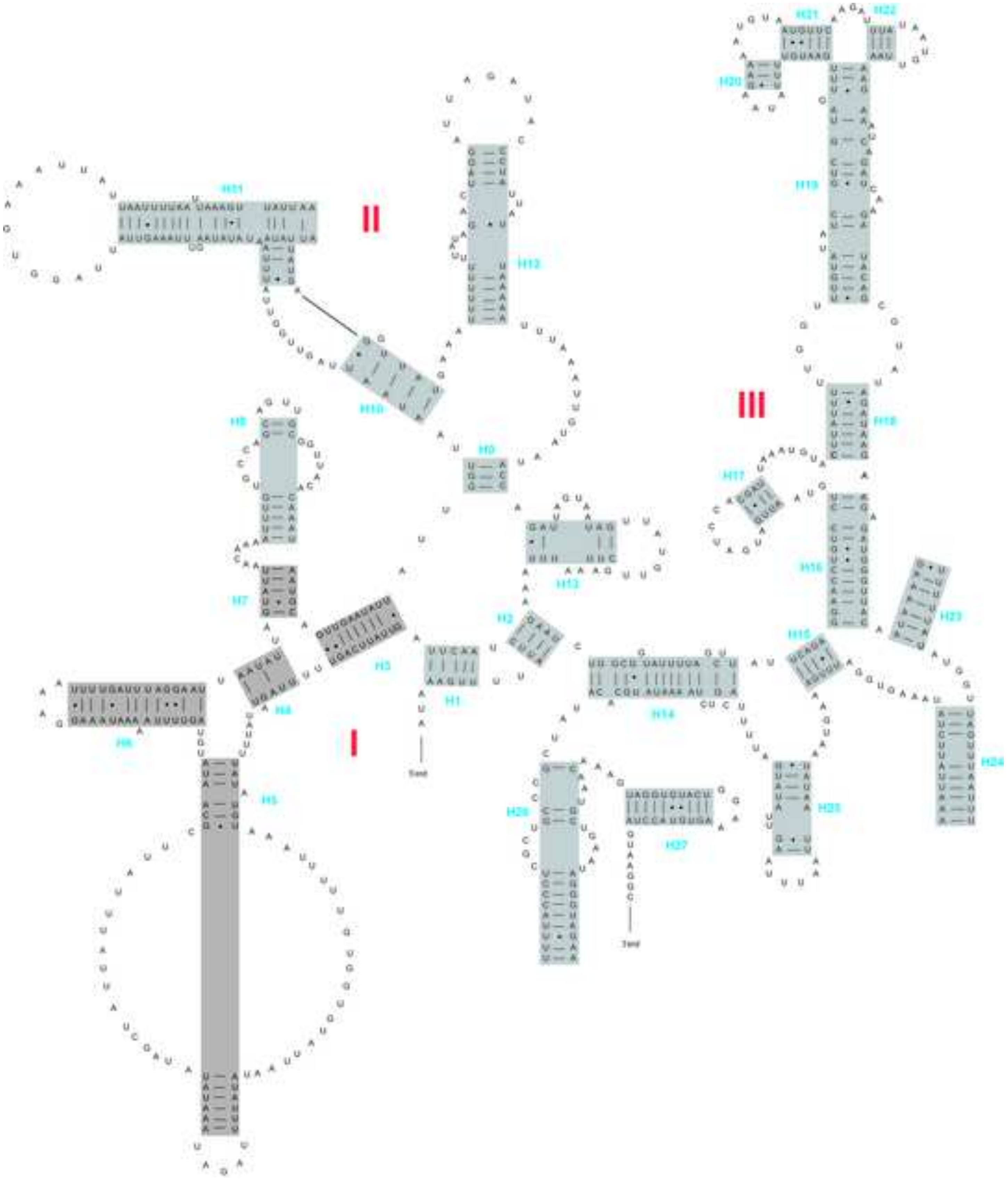

Figure 4. Phylogenetic tree inferred from the dataset PCG_aa using IQ-TREE under the models selected by ModelFinder.

Click here to access/download;Figure;Figure 4.tif

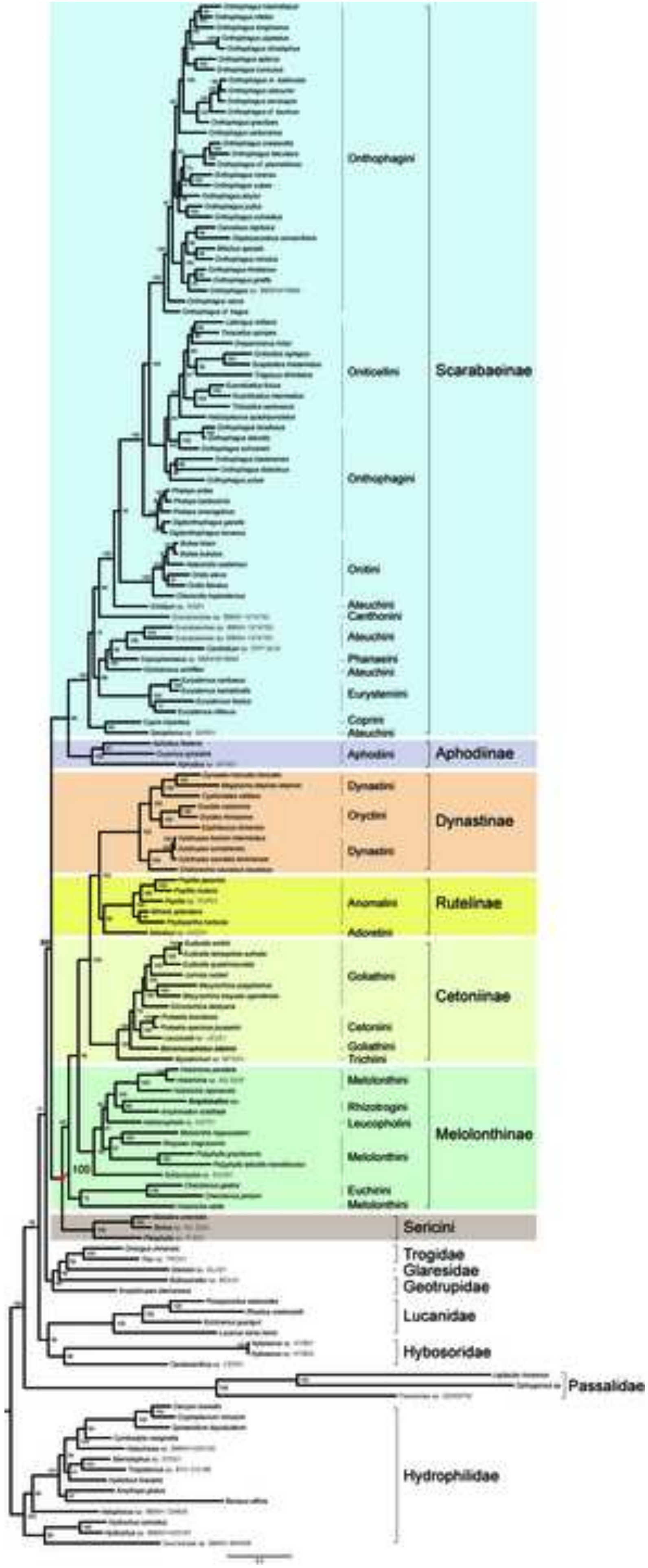

Figure 5. Phylogenetic tree inferred from the dataset PCG_aa using PhyloBayes-MPI under the model CAT-mtZOA.

Click here to access/download;Figure;Figure 5.tif

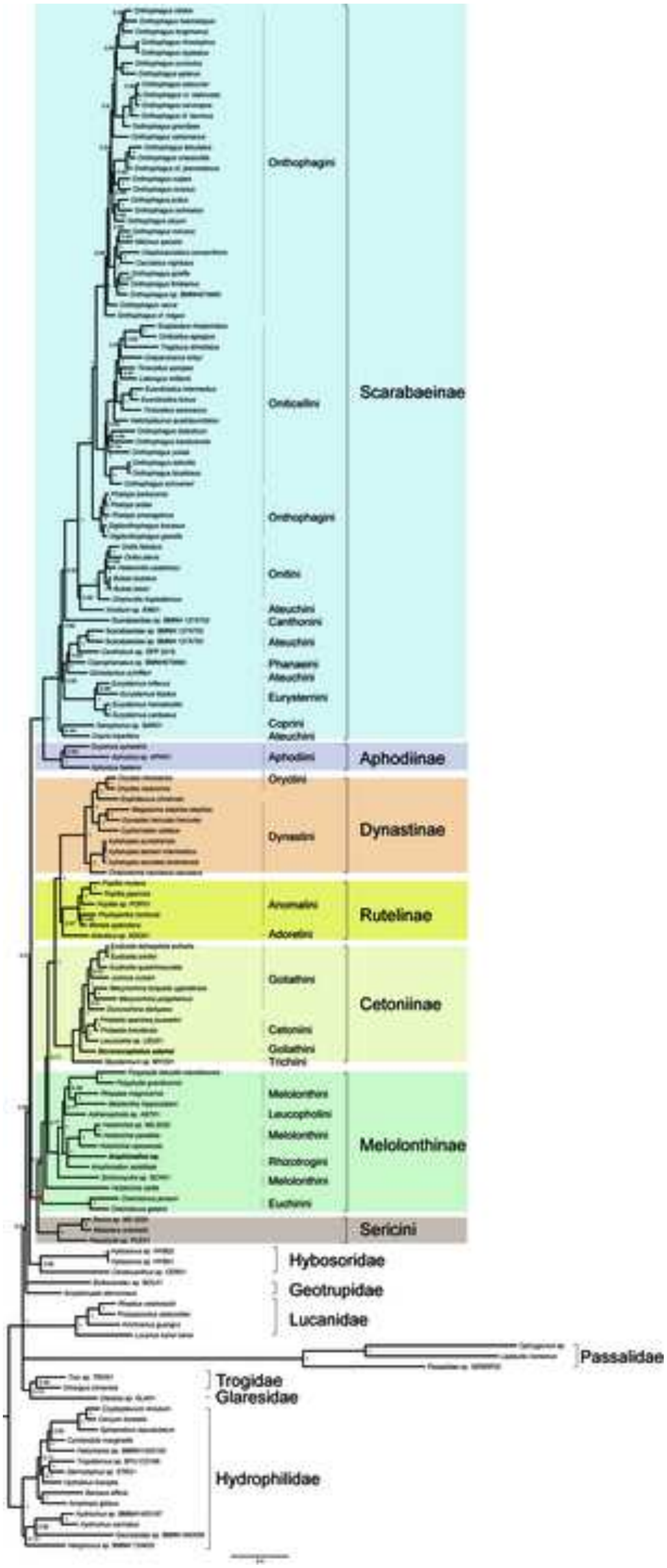

Figure 6. Phylogenetic tree inferred in IQ-TREE using the subset with 2000 sites removed from the dataset PCGRNA.

Click here to access/download;Figure;Figure 6.tif

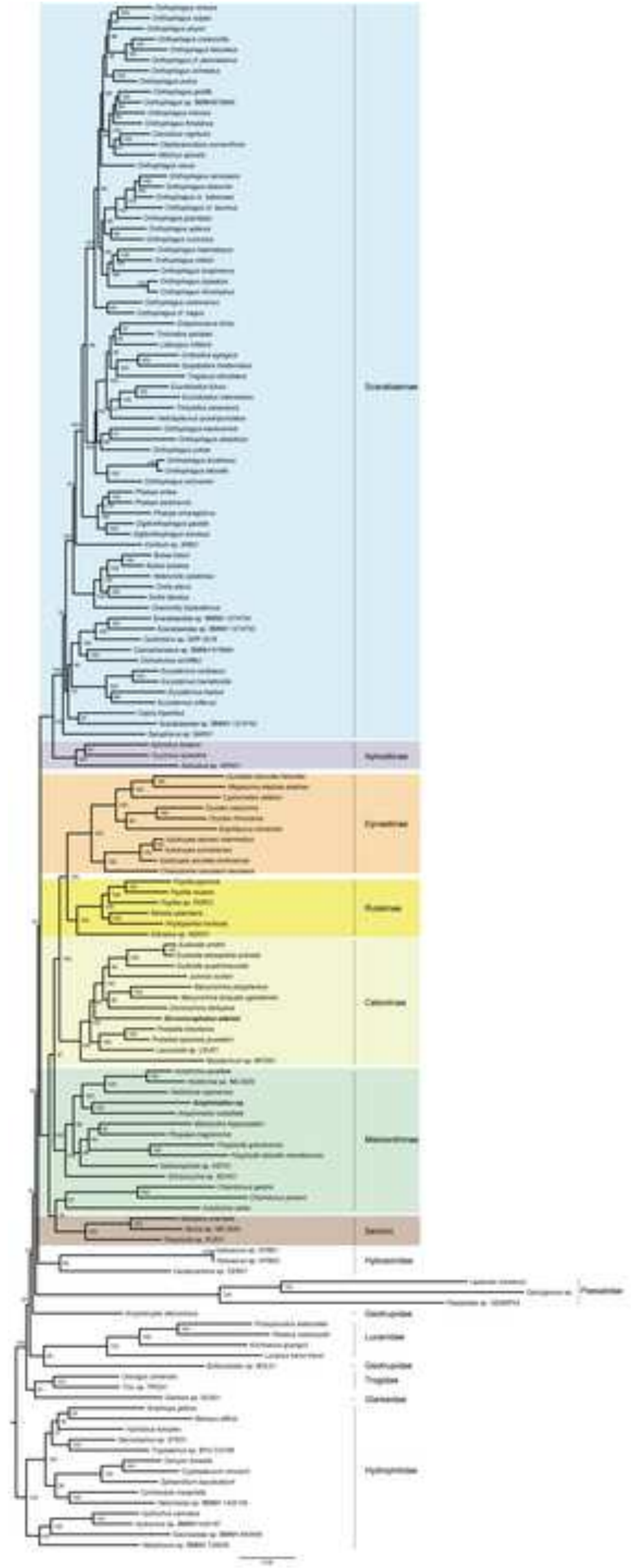

Figure 7. Results of the Four-cluster likelihood mapping (FcLM) analyses.

Click here to access/download;Figure;Figure 7.tif

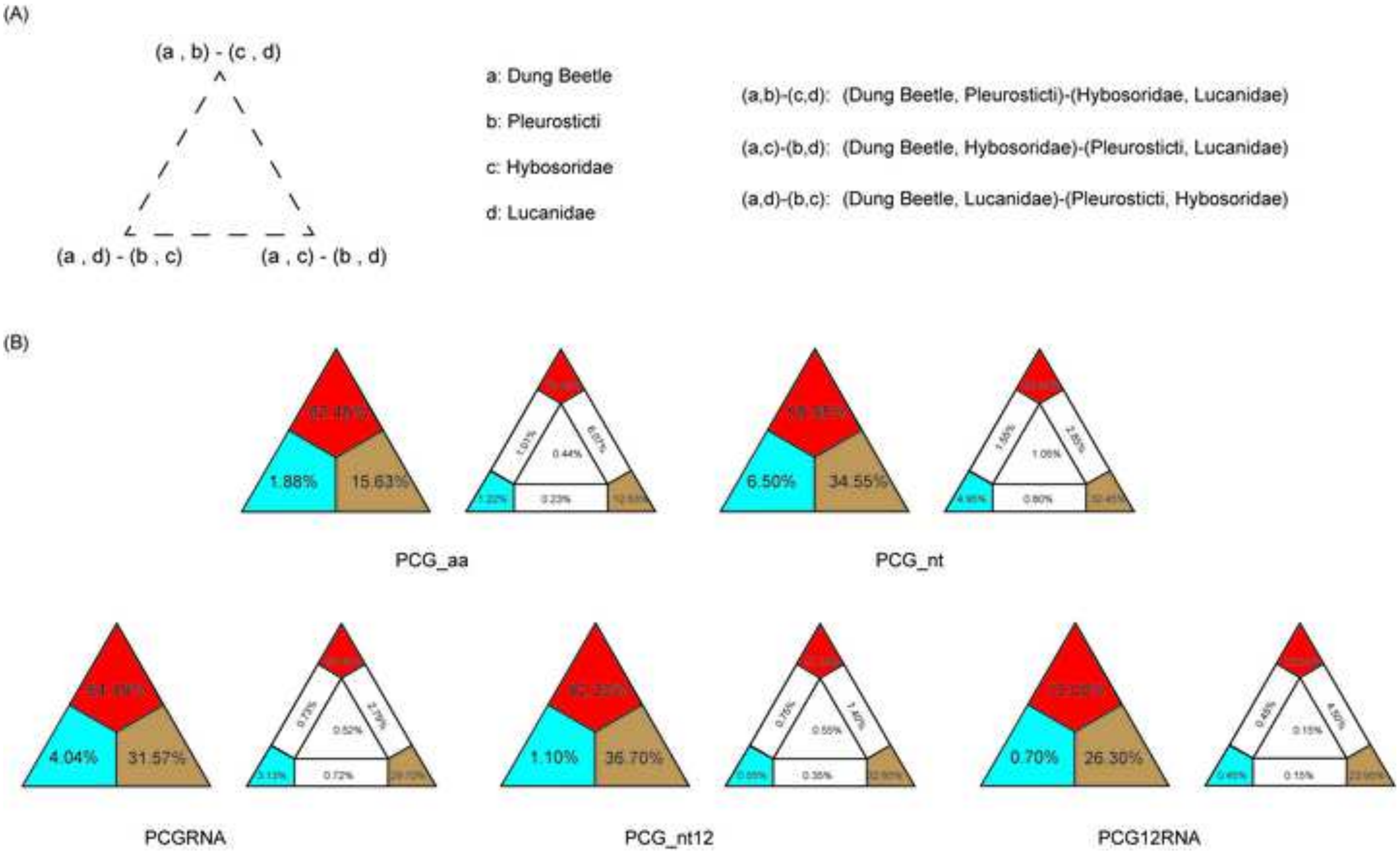

Click here to access/download
Supporting Information - Compressed/ZIP File Archive
Supporting Information files.zip

Mitochondrial phylogenomics reveals deep relationships of scarab beetles (Coleoptera, Scarabaeidae)

Shibao Guo[1*], Xingyu Lin[2], Nan Song[2*]

1 Xinyang Agriculture and Forestry University, Xinyang, Henan, China

2 College of Plant Protection, Henan Agricultural University, Zhengzhou, Henan, China

* Corresponding Authors:

Shibao Guo, Nan Song

E-mail: sbguo510@163.com; songnan@henau.edu.cn

Abstract

In this study, we newly sequenced the complete mitochondrial genomes (mitogenomes) of two phytophagous scarab beetles, and investigated the deep level relationships within Scarabaeidae combined with other published beetle mitogenome sequences. The complete mitogenomes of *Dicronocephalus adamsi* Pascoe (Cetoniinae) and *Amphimallon* sp. (Melolonthinae) are 15,563 bp and 17,433 bp in size, respectively. Both mitogenomes have the typical set of 37 genes (13 protein-coding genes, 22 transfer RNA genes, two ribosomal RNA genes) and an A+T-rich region, with the same gene arrangement found in the majority of beetles. The secondary structures for ribosomal RNA genes (*rrnL* and *rrnS*) were inferred by comparative analysis method. Results from phylogenetic analyses provide support for major lineages and current classification of Scarabaeidae. Amino acid data recovered Scarabaeidae as monophyletic. The Scarabaeidae was split into two clades. One clade contained the subfamilies Scarabaeinae and Aphodiinae. The other major clade contained the subfamilies Dynastinae, Rutelinae, Cetoniinae, Melolonthinae and Sericini. The monophyly of Scarabaeinae, Aphodiinae, Dynastinae, Cetoniinae and Sericini were strongly supported. The Scarabaeinae was the sister group of Aphodiinae. The Cetoniinae was sister to the Dynastinae + Rutelinae clade. The Melolonthinae was a non-monophyletic group. The removal of fast-evolving sites from nucleotide dataset using a pattern sorting method (OV-sorting) supported the family Scarabaeidae as a monophyletic group. At the tribe level, the Onthophagini was non-monophyletic with respect to Oniticellini. Ateuchini was sister to a large clade comprising the tribes Onthophagini, Oniticellini and Onitini. Eurysternini was a sister group of the Phanaeini + Ateuchini clade.

Keywords: Cetoniinae, Melolonthinae; mitochondrial genome; phylogeny

Introduction

The family Scarabaeidae is one of the largest and most diverse groups of beetles, with more than 30,000 described species worldwide [1-5]. According to feeding preferences, this family was divided into two major groups, namely the saprophagous~~coprophagous~~ and phytophagous lineages. ~~The~~ Most of the saprophagous scarabs~~coprophagous Scarabaeidae~~ are specialist dung feeders in adults~~ often called dung beetles~~, which are often called dung beetles and well known to most people due to their relatively large body size, significantly ecological importance and the sacred symbols of *Scarabaeus* in ancient Egypt. Dung beetles are one of the primary utilizers of mammalian dung on Earth. Many species of phytophagous scarab beetles are economically damaging pests because of the high levels of herbivory. For example, the Japanese beetle *Popillia japonica* is broadly invasive and causes millions of dollars of damage worldwide [6-8].

Despite their enormous ecological and economic importance, the phylogenetic relationships within Scarabaeidae remain controversial. The saprophagous~~dung~~ scarabs comprise two major subfamily groups~~; (~~, Scarabaeinae and Aphodiinae [9]~~)~~. The former are predominantly specialist dung feeders~~[1, 9]~~, while the latter are generalists feeding on dead or decaying matter like leaf litter and rotting fruits [1, 9]. In addition, some species classified in Hybosoridae, Ochodaeidae, Geotrupidae and other Scarabaeoidea groups were once considered to be the close relatives of Scarabaeinae and Aphodiinae, and all of them constituted the Laparosticti [10]. The phytophagous scarab beetles are traditionally grouped into four main subfamilies (Melolonthinae, Cetoniinae, Dynastinae, and Rutelinae) [11-13]. The phytophagous scarabs are called as Pleurosticti [14]. The Laparosticti and Pleurosticti are often characterized by location of the spiracles [15, 16], but see [17]. The division of Scarabaeoidea into Laparosticti and Pleurosticti is currently considered as untenable. Besides the six main subfamilies mentioned above, a few other much smaller subfamilies were recognized in the Scarabaeidae, such as Aclopinae, Allidiostomatinae, Euchirinae, Orphninae and Pachypodinae [12, 18].

The monophyly of the family Scarabaeidae as a whole and the relationships among

Formatted: Font color: Auto

Formatted: Font color: Auto

Formatted: Font color: Red

Formatted: Font color: Auto

Formatted: Not Highlight

the subfamilies have been contentious. Grebennikov and Scholtz (2004) [18] used larval morphological characters to infer the higher-level relationships of Scarabaeoidea. Their results did not support the monophyly of Scarabaeidae. The subfamilies Scarabaeinae and Aphodiinae was recovered as separate clades [18]. In contrast, the molecular phylogenetic study using the 28S rDNA and 18S rDNA sequence data found strong support for the family Scarabaeidae [11]. The monophyly of the dung beetle clade (Scarabaeinae + Aphodiinae) was also supported [11]. A phylogenetic analysis of the Coleoptera using 95 protein-coding genes also supported the monophyly of Scarabaeidae [19]. The phylogenetic analyses based on the mitochondrial genome (mitogenome) sequences consistently recovered a monophyletic Scarabaeidae [3, 20], though with the limited sampling of Scarabaeoidea. However, a phylogenetic analysis of Scarabaeoidea based on four DNA markers (the 18S rDNA and 28S rDNA genes, and the mitochondrial *cox1* and *rrnL* genes) recovered a non-monophyletic Scarabaeidae [21]. Besides the question of Scarabaeidae monophyly, the subfamilies Aphodiinae and Melolonthinae were also questioned in the previous studies [3, 11]. In fact, the classification for the Scarabaeidae differs between systematists and regions. For example, Ahrens often recognized Sericini to be a tribe of Melolonthinae (e.g., [13, 22, 23]). The tribe Sericini as a member of Melolonthinae was also supported by the study based on the 18S and 28S rDNA sequence data [12]. However, Ahrens et al (2014) recovered Melolonthinae to be a paraphyletic assemblage, with Sericini being a separate clade [21]. Several recent phylogenetic analyses based on mitogenomes often recovered sericine chafers outside Melolonthinae, and tended to support the subfamily status of Sericinae in Scarabaeidae [3, 5, 24].

The main aims of the present study are threefold: (i) to test the monophyly of Scarabaeidae; (ii) to elucidate the phylogenetic relationships among the subfamilies within Scarabaeidae; (iii) to explore the utility of the mitogenome sequence data for these phylogenetic relationships. To perform this study, we sequenced two new mitogenomes from the Cetoniinae (*Dicronocephalus adamsi* Pascoe, 1863) and the Melolonthinae (*Amphimallon* sp.). Combined with the published mitogenomes of

Scarabaeoidea construct the most comprehensive datasets of the scarab mitogenome sequences to date. DNA sequence data were analyzed under various inference methods and different data recoding schemes.

Material and methods

Ethics statement

No specific permits were required for the insect specimens collected for this study. These specimens were collected on the field of Zhengzhou. The field studies did not involve endangered or protected species. The two insect species sequenced are all common beetle species in China and are not included in the ‘‘List of Protected Animals in China”.

Taxonomic sampling

To evaluate the monophyly of Scarabaeidae and the phylogenetic relationships within this family, we chose a set of ingroup and outgroup species satisfying the following conditions: (i) inclusion of members of several major subfamilies, which ensured covering the major clades of the dung scarabs and the phytophagous scarabs; (ii) inclusion of major families within the superfamily Scarabaeoidea; (iii) choosing the Hydrophilidae to root the tree. A total of 118 ingroup species were included: 70 species from Scarabaeinae, three from Aphodiinae, ten from Dynastinae, six from Rutelinae, 12 from Cetoniinae, 14 from Melolonthinae, and three from Sericini (or Sericinae) (Table S1). Outgroup taxa for Scarabaeidae belonged to the closely-related families Trogidae (two species), Glaresidae (one species), Geotrupidae (two species), Lucanidae (four species), Hybosoridae (three species), and Passalidae (three species). The more distantly related Hydrophilidae (14 species) were also used to be the non-Scarabaeoidea outgroups. In this study, the classification within Scarabaeoidea followed those of Smith (2006) [25].

Three mitogenome sequences downloaded from the public database GenBank

(Scarabaeidae sp. BMNH 1274750, Scarabaeidae sp. BMNH 1274752, Scarabaeidae sp. BMNH 1274753). Three sequences were originally classified at the family level. We re-conducted molecular identification using the mitochondrial *cox1* gene. In addition, combined with the resulting trees inferred in the present study, we assigned Scarabaeidae sp. BMNH 1274752 to the tribe Canthonini (Scarabaeinae) and both Scarabaeidae sp. BMNH 1274750 and Scarabaeidae sp. BMNH 1274753 to the tribe Ateuchini (Scarabaeinae).

Sequencing of complete mitogenomes

Specimen collection and DNA extraction

The adult specimens of *D. adamsi* and *Amphimallon* sp. were collected at Zhengzhou, Henan province, China, in July 2019 and 2020, respectively. The specimens were preserved in absolute ethanol and stored at -80 ℃until DNA extraction. Total genomic DNA was extracted from leg muscle tissue using the TIANamp Genomic DNA Kit (TIANGEN BIOTECH CO., LTD), following the manufacturer’s protocol.

Next generation sequencing

The extracts were delivered to BGI-Shenzhen, China for library preparation and sequencing. Libraries for sequencing were prepared from cDNA, which was sheared to produce 300-4000 bp double stranded fragments. Sequencing was performed by using the BGISEQ-500 platform, with the strategy of 100 paired-end. Low quality sequencing reads were trimmed by using NGS QC Toolkit [26]. The remaining clean data (15,947,554 reads for *D. adamsi* and 21,808,396 reads for *Amphimallon* sp.) were then assembled into contigs.

Mitogenomes assembly, annotation and analysis

Clean reads were used for *de novo* assembly of mitogneomes of using GetOrganelle

v1.7.5.2 [27]. We used the GetOrganelle animal database (-F animal_mt) to identify, filter and assemble target-associated reads.

The mitogenome contigs were initially annotated in the MITOS webserver [28], under the Metazoan RefSeq 63 set and default settings. The gene boundaries of protein-coding genes were refined by alignment with closely related scarab species. Transfer RNA (tRNA) genes were determined and the corresponding secondary structures were inferred in MITOS web [28]. Secondary structures for *rrnL* and *rrnS* genes were predicted by the comparative method, with reference to the scarab beetle *Popillia mutans* [3]. The new mitogenome sequences of *D. adamsi* and *Amphimallon* sp. have been deposited at GenBank, with the accession number of MZ955033 and ON529251.

Nucleotide compositions of the mitogenome sequences were calculated using MEGA X [29]. The strand compositional bias was examined by using AT and GC-skew values calculated by the formula: AT-skew = (A-T)/(A+T) and GC-skew = (G-C)/(G+C) [30].

Multiple sequence alignment

Sequence alignments of protein-coding genes were accomplished using TranslatorX [31], with the MAFFT algorithm [32]. The poorly aligned regions and gaps were removed by ~~trimAL~~ trimAl v1.4 [33], with the "-automated1 heuristic". Ribosomal and transfer RNA genes were aligned individually in MAFFT version 7 [32], with the iterative refinement option of E-INS-i. The resulting alignments were also trimmed using trimAl v1.4 [33], with the above-mentioned parameter settings. Alignments were concatenated with FASconCAT-G_v1.04 [34] to compile the following three types of datasets: 1) PCG_nt, the concatenated nucleotide sequences including 13 protein-coding genes; 2) PCG_aa, the concatenated amino acid sequences including 13 protein-coding genes; 3) PCGRNA, the concatenated nucleotide sequences including 13 protein-coding genes, two rRNA genes and 22 tRNA genes; 4) PCG_nt12, the concatenated nucleotide sequences of 13 protein-coding genes with the third codon positions removed; and 5) PCG12RNA, the concatenated nucleotide sequences of

PCG_nt12, two rRNA genes and 22 tRNA genes.

Phylogenetic analyses

Tree searches

We employed both Maximum likelihood (ML) and Bayesian inference (BI) methods to conduct phylogenetic tree reconstructions. Model selection was conducted in ModelFinder [35] for every concatenated matrix used herein, with the alignment partitioned by gene.

ML trees were reconstructed from each of the matrices with IQ-TREE 1.6.12 [36] using the best-scoring substitution model for each gene partition (Table S2) as selected with ModelFinder [35]. Support for trees was assessed with 10,000 ultrafast bootstrap (BS) replicates [37].

Bayesian tree searches were performed with PhyloBayes-MPI 1.8 [38, 39]. For each dataset, we conducted two independent runs. The CAT-GTR model was used in the analyses of nucleotide alignments, while the CAT-mtZOA model was used in the analysis of amino acid alignment. Each run involved two chains, with a total length of 10,000 cycles. Convergence between chains was evaluated by examining the difference in frequency for all their bipartitions (maxdiff < 0.3). The consensus tree was built from the two runs with the *bpcomp* program discarding the first 1,000 trees as burn-in. Branch support was assessed by the clade posterior probabilities (PP).

We tested how slow and fast evolving sites could affect phylogenetic discordance. To do so, trees were generated from data matrices produced using the OV-sorting method [40]. This method ranks the originally concatenated alignment from the most variable sites to the most conserved sites based on the measurement of “observed variability” (OV) of each alignment position. Previous studies have suggested that accuracy of phylogenetic inference can be improved by the removal of the most variable sites from datasets using the OV-sorting method [41-43]. A series of shortened alignments were created by sequentially removing fast-evolving sites in 250 increments

from the 13,858 sites of the PCGRNA dataset. The OV-sorted datasets were subjected to unpartitioned ML analyses, with the parameter settings as described above.

Hypothesis Testing

The specific phylogenetic hypothesis, namely the relationships of major clades of Scarabaeidae, was further assessed by means of the four-cluster likelihood-mapping (FcLM) approach implemented in IQ-TREE 1.6.12 [36]. For the three datasets (PCG_nt, PCG_aa and PCGRNA), we used the same partition schemes and the corresponding models selected during the IQ-TREE tree search.

Results

Sequencing of mitochondrial genomes

Genome sequencing produced 15,947,554 and 21,808,396 paired-end reads for *D. adamsi* and *Amphimallon* sp., respectively. The approximately 3.53% and 0.13% of the reads resembled mitochondrial sequences. The 15,563 bp and 17,433 bp contigs were assembled for the mitogenomes of *D. adamsi* and *Amphimallon* sp.. Mean coverage of the two contigs reached 4339-fold and 266-fold.

The mitogneomes of *D. adamsi* and *Amphimallon* sp. have the typical set of 37 mitochondrial genes (13 protein-coding genes, 22 tRNA genes, and two rRNA genes) and a major noncoding region (also called control region or A+T-rich region). Both mitogenomes have the similar gene content and genome organization found in the majority of beetles. Nucleotide composition of the mitogenomes was A+T biased, 74.4% for *D. adamsi* and 73.5% for *Amphimallon* sp., respectively. For the majority strand in two scarab species, the AT-skew is positive (higher A than T content, 0.025 for *D. adamsi* and 0.056 for *Amphimallon* sp.) while the GC-skew is negative (higher C than G content, -0.27 for *D. adamsi* and -0.25 for *Amphimallon* sp.).

The complete set of 22 tRNA genes typical of insect mitogenomes [44] is present

in the two mitogenomes. The length of tRNA genes range from 62 bp to 71 bp. All tRNA gene sequences can be folded into the cloverleaf structure, except for *trnS1* in both species and *trnW* in *D. adamsi* (Figure 1 and Figure S1). The *trnS1* in both species and the *trnW* in *D. adamsi* have an unpaired stretch of 12 nucleotides or 7 nucleotides instead of the DHU arm.

The lengths of *rrnS* genes are 780 bp and 785 bp for *D. adamsi* and *Amphimallon* sp., and the lengths of *rrnL* genes are 1,303 bp and 1,329 bp, respectively. The secondary structures inferred for the *rrnL* and *rrnS* genes were similar to the secondary structure models proposed for other scarab beetles (e.g., *P. mutans*, [3]). Both *rrnL* genes' secondary structures include five major domains (I, II, IV, V and VI). The sequence spanning domain II and domain IV is very short that leads to a loss of the domain III (Figure 2 and Figure S 2). This pattern is often found in insect *rrnL* gene sequences. Five domains are composed of 41-42 helices. *rrnS* genes have three domains (I, II and III) composed of 26 helices (Figure 3 and Figure S3).

Phylogenetic inferences

The same alignment produced the same topology regardless of inference method used. In the ML and BI trees from the amino acid dataset PCG_aa (Figure 4-5), the family Scarabaeidae was well supported and split into two major clades. One clade contained the subfamilies Scarabaeinae and Aphodiinae, another included the subfamilies Dynastinae, Rutelinae, Cetoniinae, Melolonthinae and the tribe Sericini. The monophyly of Scarabaeinae, Aphodiinae, Dynastinae, Cetoniinae and Sericini were strongly supported (BS = 100, PP = 1). The node for Rutelinae received the statistically significant support (PP = 0.97) in the BI analysis, but not in the ML analysis (BS = 56). The Melolonthinae was non-monophyletic in both ML and BI trees. In the analyses based on the amino acid dataset, several sister-group relationships were consistently recovered with strong support. The Scarabaeinae was the sister group of Aphodiinae (BS = 100, PP = 1). The Cetoniinae was sister group to the Dynastinae + Rutelinae clade.

The analyses from the nucleotide datasets (PCG_nt and PCGRNA, Figure S4-S7) yielded tree topologies with the largely identical subfamily relationships in Scarabaeidae. The Scarabaeidae was subdivided into two major clades, which had the same composition and internal arrangement of the subfamilies to those of the analyses from the amino acid dataset. However, the family Scarabaeidae was non-monophyletic with respect to Hybosoridae and/or Lucanidae. The subfamily Rutelinae was recovered as a monophyletic group in the nucleotide analyses. The Melolonthinae was paraphyletic, with the grouping of Cheirotonus + Holotrichia as a separate clade.

Removal of third codon positions did not change tree topological structure in the nucleotide analyses. In the ML tree from the PCG12RNA dataset (Figure S8), the outgroups (Lucanidae + (Passalidae + Hybosoridae)) was sister to the phytophagous scarab clade, which resulted in a non-monophyletic Scarabaeidae. A similar branching pattern was recovered in the ML analysis of the PCG_nt12 dataset (Figure S9). In the BI tree from the PCG12RNA dataset (Figure S10), a sister group relationship between the dung scarab clade (Scarabaeinae + Aphodiinae) and Hybosoridae (PP = 0.8) led to the paraphyletic Scarabaeidae. For the PhyloBayes analysis of the dataset PCG_nt12 (Figure S11), ambiguity represented by polytomies was found in the relationships among the dung scarab clade, the phytophagous scarab clade and the two outgroup families of Passalidae and Hybosoridae. This pattern showed that removing third codon positions resulted in the loss of phylogenetic signal for resolving the deep relationships among the groups. Despite the non-monophyly of Scarabaeidae, terminal relationships among subfamilies of the family were generally stable across analyses of nucleotide datasets.

Sequentially removing the fast-evolving sites from the PCGRNA dataset produced a series of shortened alignments, which contained increasing proportion of conserved sites. As illustrated in Figure 6, the non-phylogenetic Scarabaeidae was retrieved while the 2,000 most variable sites were included in the ML analysis. After these sites were removed, the monophyletic Scarabaeidae was recovered until 5,000 sites were removed. The nodal support value for Scarabaeidae gradually increased from the analysis of the

subset containing 11,608 positions to the analysis of the subset containing 9,608 positions. In the ML trees from the subsets further removing the fast-evolving sites in the subsequent three steps, relationships within Scarabaeidae collapsed due to information loss. After this point (subset containing 8,858 positions), the Scarabaeidae was recovered to be non-monophyletic again and the entire relationships on the tree were unresolved.

The FcLM results (Figure 7), showed support for the sister-group relationship between the Scarabaeinae + Aphodiinae clade and the clade including all other scarab subfamilies (58.5% - 79.4%), which is congruent with the ML topology inferred from the amino acid dataset. Alternative relationships had poor support: the Scarabaeinae + Aphodiinae clade being sister group to Hybosoridae (14.5% - 31.8%), and the clade including Dynastinae, Rutelinae, Cetoniinae, Melolonthinae and Sericini being sister group to Hybosoridae (2.6% - 5.7%). The FcML results suggested that the monophyletic Scarabaeidae was indeed better supported by Maximum likelihood.

Discussion

The monophyly of Scarabaeidae

Traditionally, based on morphological evidence, the scarab dung beetles and the pleurostict chafers are considered to be sister groups, both of which constitute the monophyletic Scarabaeidae [45-48]. The Scarabaeidae was also recovered in recent molecular studies [19, 20, 49]. However, the Scarabaeidae was not supported by other analyses based on morphological characters [18, 50], multi-locus sequence data [21, 51-53] and single-copy nuclear protein-coding gene data [54]. In the present study, the monophyly of Scarabaeidae was strongly confirmed by ML analysis (BS = 88) based on amino acid data. In the BI analysis based on the same dataset, the Scarabaeidae was recovered, but with only weak support (PP = 0.86).

Analysis based on the nucleotide subsets produced by the OV-sorting methods showed that removal of the most variable sites has a significant effect on the

phylogenetic inference of relationships of Scarabaeidae. The subsets including more fast-evolving sites supported Scarabaeidae to be a non-monophyletic group. Whereas, the subsets containing a relatively large number of conserved positions can recover a monophyletic Scarabaeidae.

Overall, we found that all strongly supported nodes in Scarabaeidae were congruent between topologies from the amino acid dataset and the OV-sorted alignments containing more conserved positions. However, the amino acid dataset and the nucleotide dataset with full positions produced conflict result: the former recovered a monophyletic Scarabaeidae, but the latter retrieved a non-monophyletic Scarabaeidae. The possible explanation for the topological difference between phylograms may be base compositional heterogeneity, substitution model misspecification and the potential tree-building artifacts (e.g., long-branch attraction). The FcLM analyses suggested that a sister-group relationship between the dung scarab clade and the phytophagous scarab clade is better supported by ML.

The interrelationships among subfamilies within Scarabaeidae

Despite the distinct branching pattern on the monophyly of Scarabaeidae inferred from the amino acid dataset and the nucleotide dataset with full positions, both types of data provided the supported phylogenetic hypotheses in splitting Scarabaeidae into two major clades and the interrelationships between subfamilies in each of the clades.

With regard to the dung beetle clade, all analyses recovered the reciprocal monophyly of Scarabaeinae and Aphodiinae. Moreover, the sister group relationship of Scarabaeinae and Aphodiinae was strongly supported (BS = 100, PP = 1). This result was highly concordant with previous morphological [46, 50] and molecular studies [11, 20, 51, 53], but contrasted with the studies of Grebennikov and Scholtz (2004) [6] and Hunt et al. (2007) [52].

The phytophagous scarab clade has long been recognized as a monophyletic group. The earlier studies based on the morphological characters provided strong support for the clade [46, 48, 55]. This clade was further supported by the subsequent analyses

based on molecular data [11, 20, 21, 51]. However, the relationships among the subfamilies within the clade remained unstable. In our analyses based on the mitogenomic data, the monophyly of the phytophagous scarab clade was strongly supported (BS = 100, PP = 1). Moreover, we recovered a well-resolved topology with several strongly supported subfamily groups within the clade. The Sericini were unequivocally recovered as sister to the remaining pleurosticts. A sister group relationship between Dynastinae and Rutelinae was confirmed. This arrangement was in accordance with the previous morphological [46, 48, 56] and molecular analyses [11, 24]. Both Dynastinae and Rutelinae formed the sister group of Cetoniinae. This branching order was consistent with that in the study of Ahrens et al. (2014)[21].

~~*The monophyly of Rutelinae.*~~ In this study, only six mitogenome sequences were included for the Rutelinae due to the data availability of the subfamily. These sequences are from four genera classified in two tribes: the *Adoretus* of Adoretini, and the *Mimela*, *Phyllopertha* and *Popillia* of Anomalini. All analyses clustered the six Rutelinae species in a clade, with Adoretini as a sister group of Anomalini. However, in some analyses (ML PCG_aa, ML PCG_nt, BI PCG_nt, BI PCGRNA, and BI PCG_nt12), there was weak support for a Adoretini + Anomalini sister-group relationship (BS < 70, PP < 0.9). In ~~the~~ two prior studies of [9, 21], ~~the~~ Rutelinae were recovered as a paraphyletic group. Based on the analyses of four DNA markers (*18S*, *28S*, *16S*, and *cox1*), Ahrens et al. (2014) [21] recovered Adoretini as a sister group of a clade composed of Pachydemini and Dynastinae.

Gunter et al. (2016) [9] used the different molecular data (*28S*, *16S*, *12S*, and *cox1*) to yield a similar result to Ahrens et al. (2014) [21]. Rutelinae was recovered as a paraphyletic grade, with Adoretini being sister to Dynastinae [21]. The Anoplognathini and the Rutelini + Anomalini clade formed the independent lineages [21], respectively. Compared with the previous studies [9, 21], our taxon sampling for Rutelinae was incomplete. Increased taxon sampling is expected to generate a more conclusive estimate on the monophyly of Rutelinae.

Formatted: Font: Italic

Formatted: Font: Italic

Formatted: Font: Italic

Formatted: Font: Italic

Formatted: Font: Italic

Formatted: Font: Italic

Formatted: Font: Italic

Formatted: Font: Italic

Formatted: Font: Italic

Formatted: Font: 12 pt, Not Bold, Font color: Auto

Formatted: Font: Italic

Based on our preliminary analyses, Geotrupidae (including Bolboceratinae), Passalidae, and Pleocomidae form a weakly supported clade, but the exact relationships between the taxa in these families is inconclusive

In some of our preliminary analyses there is weak support for a Hybosoridae + Ochodaeidae sister-group relationship

where sampling is incomplete

The Melolonthinae was recovered to be a paraphyletic group. In fact, the monophyly of Melolonthinae has been questioned in previous studies. Ahrens et al. (2014) [21] recovered Melolonthinae as a paraphyletic group based on the analysis of multi-locus data. A prior mitogenomic analysis also supported Melolonthinae as a paraphyletic assemblage [3].

Tribal relationships within Scarabaeinae and implications for nesting behaviors

Dung beetles in Scarabaeinae were once classified into two main groups based on their rolling and tunneling behavior, namely Scarabaeinae and Coprinae [57]. The former group included six tribes Eurysternini, Deltochilini, Eucraniini, Gymnopleurini, Scarabaeini and Sisyphini, and the latter contained six tribes Coprini, Oniticellini, Onitini, Onthophaghini, Ateuchini and Phanaeini. Although this system was widely accepted by the subsequent systematists on the Scarabaeinae, some questions have been raised on the classification of the subfamily. Montreuil (1998) [58] assigned the genera of Dichotomiini to the tribe Coprini and renamed the tribe Dichotomiini as Ateuchini. Scholtz et al (2009) [59] suggested that Dichotomiini may be a polyphyletic group. A

molecular phylogenetic analysis recovered the rolling Canthonini and tunnelling Dichotomiini to be polyphyletic [60]. The phylogenetic analysis based on the morphological characters further supported the polyphyly of Dichotomiini [61]. A morphological study by Philips et al (2004) [62] did not support the monophyly of the Ateuchini. Bouchard (2011) [63] revised the system of Balthasar (1963) [57] to include 11 tribes in Scarabaeinae, of which Eurysternini was considered as a subtribe in Oniticellini.

~~In our analyses, the subfamily Scarabaeinae was sampled sufficiently to discuss internal relationships~~For the tribal relationships within Scarabaeinae, our results are consistent with the study of Breeschoten et al. (2016) [64]. ~~Based on the current mitogenomic data, t~~The Onthophagini with the extensive taxon sampling was a non-monophyletic group. The monophyletic Oniticellini was nested within a large clade composed of all members of Onthophagini. A previous morphological study has proposed to treat Oniticellini as a subgroup of Onthophagini [65]. The molecular phylogenetic analyses always placed Oniticellini within Onthophagini [48, 49]. This study based on the mitogenomic data confirmed the pattern recovered in the previous analyses [48, 49, 64, 65]. In the ML analysis of amino acid data, the Onitini was recovered as a sister group of the clade including Onthophagini and Oniticellini. A similar result was obtained in the phylogeny of previous studies [64-68].

Ateuchini represented by the species of *Xinidium* sp. was sister to a large clade comprising the tribes Onthophagini, Oniticellini and Onitini. The placement of Ateuchini is similar to that in the study of multi-locus sequence data [67]. The remaining species from Ateuchini were distributed in three clades at the base of the tree, which rendered a non-monophyletic Ateuchini. Based on the analysis of adult morphological characters, Montreuil (1998) [58] recognized Ateuchini to be non-monophyletic.

The Phanaeini represented by a single species of *Coprophanaeus* sp. BMNH679884 was placed in an intermediate position between *Dichotomius* and *Canthidium*. This placement of Phanaeini was incongruent with the previous molecular

Formatted: Font: (Default) Times New Roman, 12 pt, Font color: Auto

phylogeny inferred by Monaghan et al. (2007) [67]. In the tree of Monaghan et al. (2007) [68], the *Dichotomius* and *Canthidium* were sister groups, both of which were sister to the Phanaeini + Eucraniini clade. Due to the limited availability of mitogenome sequences, no taxon sampling of Eucraniini was included in the present study. Therefore, there is need for further sequence mitogenomes from the Eucraniini to elucidate the relationships among the tribes.

The monophyletic Eurysternini was a sister group of the clade including Phanaeini and Ateuchini. The *Sarophorus* + *Copris* clade was sister to all other Scarabaeinae lineages. The early diverging position of *Sarophorus* was congruent with Mlambo et al. (2015) [67]. However, the sister group relationship between *Sarophorus* and *Copris* was different from that in Mlambo et al. (2015) [66]. In the tree from Mlambo et al. (2015) [66], both genera had a distant relationship.

According to literatures [62, 66], the *Sarophorus* are non-dung-feeding while Coprini are tunneling. The Eurysternini and Canthonini are recognized as rollers. The tribes Dichotomiini, Phanaeini, Onitini, Oniticellini and Onthophagini and the genus *Xinidium* are often considered as tunnellers. An early phylogenetic analysis using morphological characters did not support the hypothesis of the roller/tunneller classification in Scarabaeinae [69]. In the phylogenetic framework estimated by the current mitogenome sequences, rolling and tunneling behaviors were intermixed throughout the Scarabaeinae lineages. This pattern was consistent with the prior studies [62, 66, 67].

Conclusion

In this study, we newly sequenced two mitogenomes from scarab beetles belonging to Cetoniinae and Melolonthinae. Combined with the published beetle mitogenomes, we constructed the most comprehensive mitogenome sequence datasets to date. In which, we have included all major subfamilies of Scarabaeidae. Results provide support for major lineages and current classification of Scarabaeidae. The amino acid dataset and the OV-sorted alignments containing more conserved positions supported the family

Scarabaeidae as a monophyletic group. This result was congruent with some morphological studies [45-48], but contrasted with several molecular studies [21, 51-54]. The sister group relationships between Scarabaeinae and Aphodiinae and between Dynastinae and Rutelinae were confirmed. The Melolonthinae was non-monophyletic. The present mitogenome data supported Sericini as an independent clade that indicated a subfamily rank of this group. At the tribe level, the Onthophagini was non-monophyletic with respect to Oniticellini.

Supporting Information files~~Supplementary Materials~~:

Figure S1: The secondary structures of tRNA genes inferred for *Amphimallon* sp.~~;~~ .

Figure S2: The secondary structure of *rrnL* inferred for *Amphimallon* sp.~~;~~ .

Figure S3: The secondary structure of *rrnS* inferred for *Amphimallon* sp.~~;~~ .

Figure S4: Phylogenetic tree of relationships within Scarabaeidae based on Maximum likelihood analysis of the dataset PCG_nt~~;~~.

Figure S5: Phylogenetic tree inferred from the dataset PCG_nt based on Bayesian inference~~;~~.

Figure S6: Phylogenetic tree of relationships within Scarabaeidae based on Maximum likelihood analysis of the dataset PCGRNA~~;~~.

Figure S7: Phylogenetic tree inferred from the dataset PCGRNA based on Bayesian inference~~;~~.

Figure S8: Phylogenetic tree of relationships within Scarabaeidae based on Maximum likelihood analysis of the dataset PCG12RNA.

Figure S9: Phylogenetic tree of relationships within Scarabaeidae based on Maximum likelihood analysis of the dataset PCG_nt12.

Figure S10: Phylogenetic tree inferred from the dataset PCG12RNA based on Bayesian inference.

Figure S11: Phylogenetic tree inferred from the dataset PCG_nt12 based on Bayesian inference.

Formatted: Font: Bold

Formatted: Font: Bold

Formatted: Font: Bold

Formatted: Font: Bold

Formatted: Font: Bold

Formatted: Font: Bold

Formatted: Font: Bold

Figure S8: Phylogenetic tree of relationships within Scarabaeidae based on Maximum likelihood analysis of the dataset PCG_nt12; Figure S9: Phylogenetic tree inferred from the dataset PCG_nt12 based on Bayesian inference; Figure S10: Phylogenetic tree of relationships within Scarabaeidae based on Maximum likelihood analysis of the dataset PCG12RNA; Figure S11: Phylogenetic tree inferred from the dataset PCG12RNA based on Bayesian inference; Figure S12: Tribal relationships inferred for the subfamily Scarabaeinae from the dataset PCG_aa based on (A) Maximum likelihood analysis and (B) Bayesian inference.; **Table S1**: Taxa included in this study; . **Table S2**: The partitioning schemes and best-fitting modes selected by ModelFinder for the dataset (A) PCG_nt, (B) PCG_aa, (C) PCGRNA, (D) PCG_nt12 and (E) PCG12RNA.

Author Contributions: Conceptualization, S.G. and N.S.; methodology, S.G., N.S. and X.L.; software, N.S. and X.L.; validation, S.G., N.S. and X.L.; formal analysis, N.S. and X.L.; investigation, N.S. and X.L.; resources, S.G. and N.S.; data curation, N.S. and X.L.; writing—original draft preparation, N.S.; writing—review and editing, S.G. and N.S.; visualization, S.G. and N.S.; supervision, S.G.; project administration, S.G. and N.S.; funding acquisition, S.G. and N.S. All authors have read and agreed to the published version of the manuscript.

Funding: Please add: This research was funded by the National Natural Science Foundation of China, grant number U1904104, and the Foundation of Central Laboratory of Xinyang Agriculture and Forestry University, grant number FCL202003. The funders had no role in study design, data collection and analysis, decision to publish, or preparation of the manuscript.

Data Availability Statement: The mitogenome sequences of *Dicronocephalus adamsi* and *Amphimallon* sp. have been deposited at GenBank, with the accession number of MZ955033 and ON529251.

Formatted: Font: Bold

Formatted: Font: Bold

Formatted: Font: Bold

Formatted: Font: Bold

Formatted: Font: Bold

Formatted: Font: Bold

Conflicts of Interest: The authors declare no conflict of interest.

References

1. Scholtz C, Grebennikov V. In Handbook of Zoology Vol. IV, Arthropoda, Part II, Insecta. Kristensen NP, Beutel RG. Eds. Coleoptera Vol 1, Chap 12. Walter De Gruyter, Berlin; 2005.
2. Huang S, Sheng P, Zhang H. Isolation and identification of cellulolytic bacteria from the gut of Holotrichia parallela larvae (Coleoptera: Scarabaeidae). Int J Mol Sci. 2012;13(3):2563-77.
3. Song N, Zhang H. The mitochondrial genomes of phytophagous scarab beetles and systematic implications. J Insect Sci. 2018;18(6):11.
4. Jeong JS, Kim MJ, Kim I. The mitochondrial genome of the dung beetle, Copris tripartitus, with mitogenomic comparisons within Scarabaeidae (Coleoptera). Int J Biol Macromol. 2020;144:874-91.
5. Ayivi SPG, Tong Y, Storey KB, Yu D-N, Zhang J-Y. The Mitochondrial Genomes of 18 New Pleurosticti (Coleoptera: Scarabaeidae) Exhibit a Novel trnQ-NCR-trnI-trnM Gene Rearrangement and Clarify Phylogenetic Relationships of Subfamilies within Scarabaeidae. Insects. 2021;12(11):1025.
6. Hengeveld R. Dynamics of biological invasions: Springer Science & Business Media; 1989.
7. Jameson ML, Paucar-Cabrera A, Solís A. Synopsis of the New World genera of Anomalini (Coleoptera: Scarabaeidae: Rutelinae) and description of a new genus from Costa Rica and

Nicaragua. Ann Entomol Soc Am. 2003;96(4):415-32.

8. Mori N, Santoiemma G, Glazer I, Gilioli G, Ciampitti M, Cavagna B, et al. Management of Popillia japonica in container-grown nursery stock in Italy. Phytoparasitica. 2022;50(1):83-9.

9. Gunter NL, Weir TA, Slipinksi A, Bocak L, Cameron SL. If dung beetles (Scarabaeidae: Scarabaeinae) arose in association with dinosaurs, did they also suffer a mass co-extinction at the K-Pg boundary? PloS ONE. 2016;11(5):e0153570.

10. Woodruff R. The Scarab Beetles of Florida. Pt. I. The Laparosticti. Arthropods of Florida and Neighbouring Land Areas. 1973;8:1-220.

11. Eberle J, Myburgh R, Ahrens D. The evolution of morphospace in phytophagous scarab chafers: No competition-no divergence? PloS ONE. 2014;9(5):e98536.

12. Smith AB, Hawks DC, Heraty JM. An overview of the classification and evolution of the major scarab beetle clades (Coleoptera: Scarabaeoidea) based on preliminary molecular analyses. Coleopts Bull. 2006;60(5):35-46.

13. Ahrens D, Vogler AP. Towards the phylogeny of chafers (Sericini): analysis of alignment-variable sequences and the evolution of segment numbers in the antennal club. Mol Phylogenet Evol. 2008;47(2):783-98.

14. W E. Naturgeschichte der Insecten Deutschlands. Erste Abteilung, Coleoptera. Berlin: Nicolaische Buchhandlung. 1847;9:35-46.

15. Jameson ML, Ratcliffe BC. Series Scarabaeiformia Crowson 1960, Superfamily Scarabaeoidea Latreille 1802. Papers in Entomology. 2002:50.

16. Anlaş S, Keith D, Tezcan S. Notes on the seasonal dynamics of some coprophagous Scarabaeoidea (Coleoptera) species in Manisa province, western Anatolia. Turk entomol derg.

2011;35(3):447-60.

17. Ritcher PO. Spiracles of adult Scarabaeoidea (Coleoptera) and their phylogenetic significance. I. The abdominal spiracles. Ann Entomol Soc Am. 1969;62(4):869-80.

18. Grebennikov VV, Scholtz CH. The basal phylogeny of Scarabaeoidea (Insecta: Coleoptera) inferred from larval morphology. Invertebr Syst. 2004;18(3):321-48.

19. Zhang S-Q, Che L-H, Li Y, Liang D, Pang H, Ślipiński A, et al. Evolutionary history of Coleoptera revealed by extensive sampling of genes and species. Nat Commun. 2018;9(1):1-11.

20. Timmermans MJ, Barton C, Haran J, Ahrens D, Culverwell CL, Ollikainen A, et al. Family-Level Sampling of Mitochondrial Genomes in Coleoptera: Compositional Heterogeneity and Phylogenetics. Genome Biol Evol. 2015;8(1):161-75. Epub 20151208. doi: 10.1093/gbe/evv241. PubMed PMID: 26645679; PubMed Central PMCID: PMCPMC4758238.

21. Ahrens D, Schwarzer J, Vogler AP. The evolution of scarab beetles tracks the sequential rise of angiosperms and mammals. P Roy Soc B-Biol Sci. 2014;281(1791):20141470.

22. Ahrens D. The phylogeny of Sericini and their position within the Scarabaeidae based on morphological characters (Coleoptera: Scarabaeidae). Syst Entomol. 2006;31(1):113-44.

23. Ahrens D. Taxonomic changes and an updated catalogue for the Palaearctic Sericini (Coleoptera: Scarabaeidae: Melolonthinae). Zootaxa. 2007;1504(1):1–51-1–.

24. Li Y, Nie R, Lu Y, Lee S, Zhao Z, Wu L, et al. Seven new mitochondrial genomes of phytophagous scarab beetles (Coleoptera: Scarabaeidae) and phylogenetic implications. Zootaxa. 2022;5138(3):324-38.

25. Smith AB. A review of the family-group names for the superfamily Scarabaeoidea

(Coleoptera) with corrections to nomenclature and a current classification. Coleopts Bull. 2006;60(mo5):144-204.

26. Patel RK, Jain M. NGS QC Toolkit: a toolkit for quality control of next generation sequencing data. PloS ONE. 2012;7(2):e30619.

27. Jin J-J, Yu W-B, Yang J-B, Song Y, DePamphilis CW, Yi T-S, et al. GetOrganelle: a fast and versatile toolkit for accurate de novo assembly of organelle genomes. Genome Biol. 2020;21(1):1-31.

28. Bernt M, Bleidorn C, Braband A, Dambach J, Donath A, Fritzsch G, et al. A comprehensive analysis of bilaterian mitochondrial genomes and phylogeny. Mol Phylogenet Evol. 2013;69(2):352-64.

29. Kumar S, Stecher G, Li M, Knyaz C, Tamura K. MEGA X: molecular evolutionary genetics analysis across computing platforms. Mol Biol Evol. 2018;35(6):1547.

30. Perna NT, Kocher TD. Patterns of nucleotide composition at fourfold degenerate sites of animal mitochondrial genomes. J Mol Evol. 1995;41(3):353-8.

31. Abascal F, Zardoya R, Telford MJ. TranslatorX: multiple alignment of nucleotide sequences guided by amino acid translations. Nucleic Acids Res. 2010;38(suppl_2):W7-W13.

32. Katoh K, Standley DM. MAFFT multiple sequence alignment software version 7: improvements in performance and usability. Mol Biol Evol. 2013;30(4):772-80.

33. Capella-Gutiérrez S, Silla-Martínez JM, Gabaldón T. trimAl: a tool for automated alignment trimming in large-scale phylogenetic analyses. Bioinf. 2009;25(15):1972-3.

34. Kück P, Longo GC. FASconCAT-G: extensive functions for multiple sequence alignment preparations concerning phylogenetic studies. Front Zool. 2014;11(1):1-8.

35. Kalyaanamoorthy S, Minh BQ, Wong TK, Von Haeseler A, Jermiin LS. ModelFinder: fast model selection for accurate phylogenetic estimates. Nat Methods. 2017;14(6):587-9.

36. Nguyen L-T, Schmidt HA, Von Haeseler A, Minh BQ. IQ-TREE: a fast and effective stochastic algorithm for estimating maximum-likelihood phylogenies. Mol Biol Evol. 2015;32(1):268-74.

37. Minh BQ, Nguyen MAT, von Haeseler A. Ultrafast approximation for phylogenetic bootstrap. Mol Biol Evol. 2013;30(5):1188-95.

38. Lartillot N, Philippe H. A Bayesian mixture model for across-site heterogeneities in the amino-acid replacement process. Mol Biol Evol. 2004;21(6):1095-109.

39. Lartillot N, Rodrigue N, Stubbs D, Richer J. PhyloBayes MPI: phylogenetic reconstruction with infinite mixtures of profiles in a parallel environment. Syst Biol. 2013;62(4):611-5.

40. Goremykin VV, Nikiforova SV, Bininda-Emonds OR. Automated removal of noisy data in phylogenomic analyses. J Mol Evol. 2010;71(5):319-31.

41. Goremykin VV, Nikiforova SV, Biggs PJ, Zhong B, Delange P, Martin W, et al. The evolutionary root of flowering plants. Syst Biol. 2013;62(1):50-61.

42. Parks M, Cronn R, Liston A. Separating the wheat from the chaff: mitigating the effects of noise in a plastome phylogenomic data set from Pinus L.(Pinaceae). BMC Evol Biol. 2012;12(1):1-17.

43. Zhong B, Deusch O, Goremykin VV, Penny D, Biggs PJ, Atherton RA, et al. Systematic error in seed plant phylogenomics. Genome Biol Evol. 2011;3:1340-8.

44. Cameron S. Insect mitochondrial genomics: implications for evolution and phylogeny. Annu Rev Entomol. 2014;59(1):95-117.

45. Browne D, Scholtz C. Phylogeny of the families of Scarabaeoidea (Coleoptera) based on characters of the hindwing articulation, hindwing base and wing venation. Syst Entomol. 1995;20(3):145-73.

46. Browne J, Scholtz CH. Evolution of the scarab hindwing articulation and wing base: a contribution toward the phylogeny of the Scarabaeidae (Scarabaeoidea: Coleoptera). Syst Entomol. 1998;23(4):307-26.

47. Browne J, Scholtz CH. A phylogeny of the families of Scarabaeoidea (Coleoptera). Syst Entomol. 1999;24(1):51-84.

48. Howden H. Larval and adult characters of Frickius Germain, its relationship to the Geotrupini, and a phytogeny of some major taxa in the Scarabaeoidea (Insecta: Coleoptera). Can J Zool. 1982;60(11):2713-24.

49. Mckenna DD, Farrell BD, Caterino MS, Farnum CW, Hawks DC, Maddison DR, et al. Phylogeny and evolution of Staphyliniformia and Scarabaeiformia: forest litter as a stepping stone for diversification of nonphytophagous beetles. Syst Entomol. 2015;40(1):35-60.

50. Lawrence JF, Ślipiski A, Seago AE, Thayer MK, Newton AF, Marvaldi AE, editors. Phylogeny of the Coleoptera based on morphological characters of adults and larvae. Annales zoologici; 2011: Museum and Institute of Zoology, Polish Academy of Sciences.

51. Bocak L, Barton C, Crampton-Platt A, Chesters D, Ahrens D, Vogler AP. Building the C oleoptera tree-of-life for> 8000 species: composition of public DNA data and fit with L innaean classification. Syst Entomol. 2014;39(1):97-110.

52. Hunt T, Bergsten J, Levkanicova Z, Papadopoulou A, John OS, Wild R, et al. A comprehensive phylogeny of beetles reveals the evolutionary origins of a superradiation.

Science. 2007;318(5858):1913-6.

53. Mckenna DD, Wild AL, Kanda K, Bellamy CL, Beutel RG, Caterino MS, et al. The beetle tree of life reveals that C oleoptera survived end-P ermian mass extinction to diversify during the C retaceous terrestrial revolution. Syst Entomol. 2015;40(4):835-80.

54. Cai C, Tihelka E, Giacomelli M, Lawrence JF, Ślipiński A, Kundrata R, et al. Integrated phylogenomics and fossil data illuminate the evolution of beetles. Royal Society Open Science. 2022;9(3):211771.

55. Browne D, Scholtz CH. The morphology of the hind wing articulation and wing base of the Scarabaeoidea (Coleoptera) with some phylogenetic implications: Zoologisches Forschungsinstitut und Museum Alexander Koenig; 1996.

56. Jameson ME. Phylogenetic analysis of the subtribe Rutelina and revision of the Rutela generic groups (Coleoptera: Scarabaeidae: Rutelinae: Rutelini): University of Kansas; 1997.

57. V B. Scarabaeidae und Aphodiidae der Palaearktischen und Orientalischen Region (Coleoptera, Lamellicornia) Verlag Tschechosl. Akad Wissenschaft, Prague Berlin: Nicolaische Buchhandlung. 1963.

58. Montreuil O, editor Analyse phylogénétique et paraphylie des Coprini et Dichotomiini (Coleoptera: Scarabaeidae). Scénario biogéographique. Annales de la Société entomologique de France; 1998.

59. Scholtz C DA, Kryger U. Section C. . Phylogeny of the Scarabaeinae. . Evolutionary Biology and Conservation of Dung Beetles. 2009;225-237.

60. Sole CL, Scholtz CH. Did dung beetles arise in Africa? A phylogenetic hypothesis based on five gene regions. Mol Phylogenet Evol. 2010;56(2):631-41.

61. Tarasov S, Genier F. Innovative Bayesian and parsimony phylogeny of dung beetles (Coleoptera, Scarabaeidae, Scarabaeinae) enhanced by ontology-based partitioning of morphological characters. PloS ONE. 2015;10(3):e0116671.

62. Philips TK, Pretorius E, Scholtz CH. A phylogenetic analysis of dung beetles (Scarabaeinae: Scarabaeidae): unrolling an evolutionary history. Invertebr Syst. 2004;18(1):53-88.

63. Bouchard P, Bousquet Y, Davies AE, Alonso-Zarazaga MA, Lawrence JF, Lyal CH, et al. Family-group names in Coleoptera (Insecta). ZooKeys. 2011;(88):1.

64. Breeschoten T, Doorenweerd C, Tarasov S, Vogler AP. Phylogenetics and biogeography of the dung beetle genus Onthophagus inferred from mitochondrial genomes. Mol Phylogenet Evol. 2016;105:86-95.

65. Tarasov SI, Solodovnikov AY. Phylogenetic analyses reveal reliable morphological markers to classify mega-diversity in Onthophagini dung beetles (Coleoptera: Scarabaeidae: Scarabaeinae). Cladistics. 2011;27(5):490-528.

66. Tarasov S, Dimitrov D. Multigene phylogenetic analysis redefines dung beetles relationships and classification (Coleoptera: Scarabaeidae: Scarabaeinae). BMC Evol Biol. 2016;16(1):1-19.

67. Mlambo S, Sole CL, Scholtz CH. A molecular phylogeny of the African Scarabaeinae (Coleoptera: Scarabaeidae). 2015.

68. Monaghan MT, Inward DJ, Hunt T, Vogler AP. A molecular phylogenetic analysis of the Scarabaeinae (dung beetles). Mol Phylogenet Evol. 2007;45(2):674-92.

69. Zunino M. Essai préliminaire sur l'évolution des armures génitales des Scarabaeinae, par

rapport à la taxonomie du groupe et à l'évolution du comportement de nidification [Col. Scarabaeidae]. Bulletin de la Societe entomologique de France. 1983;88(7):531-42.

Figure legends

Figure 1. The secondary structures of tRNA genes inferred for *Dicronocephalus adamsi*. Dashes in-dicate Watson-Crick base pairing. Dots indicate G-U base pairs.

Figure 2. The secondary structure of rrnL inferred for *Dicronocephalus adamsi*. Dashes indicate Watson-Crick base pairing. Dots indicate G-U base pairs. Red roman numbers denote the domains, and blue numbers denote the helices.

Figure 3. The secondary structure of rrnS inferred for *Dicronocephalus adamsi*. Dashes indicate Watson-Crick base pairing. Dots indicate G-U base pairs. Red roman numbers denote the domains, and blue numbers denote the helices.

Figure 4. Phylogenetic tree inferred from the dataset PCG_aa using IQ-TREE under the models selected by ModelFinder. In the phylogenetic tree, node numbers show the bootstrap support values. Scale bar represents substitutions/site. Bold denotes the newly sequenced species. Colors identify the subfamily groups.

Figure 5. Phylogenetic tree inferred from the dataset PCG_aa using PhyloBayes-MPI under the model CAT-mtZOA. In the phylogenetic tree, numbers around nodes indicate posterior proba-bilities. Scale bar represents substitutions/site. Bold denotes the newly sequenced species. Colors identify the subfamily groups.

Figure 6. Phylogenetic tree inferred in IQ-TREE using the subset with 2000 sites removed from the dataset PCGRNA. Node numbers show the bootstrap support values. Scale bar represents sub-stitutions/site. Colors identify the subfamily groups.

Figure 7. Results of the Four-cluster likelihood mapping (FcLM) analyses. (A) Alternative hy-potheses on the relationships of major clades of Scarabaeidae. (B) FcLM

results for the concatenated amino acid (PCG_aa) and nucleotide (PCG_nt, PCGRNA, PCG_nt12 and PCG12RNA) alignments.

Responses to comments

Dear Reviewers,

Thank you for your very careful review of our paper, and for the comments, corrections and suggestions that ensued. A major revision of the paper has been carried out to take all of them into account. And in the process, we believe the paper has been significantly improved.

In the present "Responses to comments", we sequentially address all of the points raised in each of the comments made by different reviewers.

Reviewer #1:

Q1: Breeschoten et al. 2016 for which much of the included sequences were published in,

Response: This reference has been cited in Table S1.

In the old version, we find the references associated with the mitogenome sequences from GenBank. Although much of the included sequences are from the Breeschoten et al. 2016, the references in GenBank are not updated where the state of "JOURNAL" is still "Unpublished". According to your comment, we read the paper of Breeschoten et al. 2016 and checked the mitogenome sequences included in it. Indeed, much of the included sequences come from this paper.

Thank you for this comment.

Q2: phylogenies that address the monophyly of the Scarabaeidae including McKenna et al. 2014 and Gunter et al. 2016 especially the most recent papers with dense gene or taxon sampling- are

relatively well resolved and provide a broader snap-shot of the deep level relationship that this paper attempts to address.

Response: After careful inspection, we found that McKenna et al. 2014 you mentioned might be the one of McKenna et al. 2015 (please see details in the following).

McKenna DD, Farrell BD, Caterino MS, Farnum CW, Hawks DC, Maddison DR, et al. Phylogeny and evolution of Staphyliniformia and Scarabaeiformia: forest litter as a stepping-stone for diversification of non-phytophagous beetles. Syst. Entomol. 2015; 40: 35–60. doi: 10.1111/syen.12093

This paper included the content of addressing the monophyly of the Scarabaeidae. This reference has been cited in this version. Page 12 Line 303, and Page 15 Lines 395-396.

In addition, we also read carefully the paper of Gunter et al. 2016 and cited it in this version. Page 3 Lines 52, 55 and Page 14 Lines 356-360.

Q3: It is extremely surprising to me that the Lucanidae rendered Scarabaeidae para-phyletic in some analysis. It is generally considered one of the earliest branching members of the superfamily as supported by molecular result and the deep fossil record. This surprising result may indicate that the mt-genome phylogeny is either insufficiently sampled at a taxon-level or is inappropriate for resolving relationships. When analyzed by amino acids and removing the 3rd codon results were more congruent with past analyses.

Response: I am sorry that this is a mistake in writing. In the preliminary analysis on the datasets with the different taxon sampling, some resulting trees might please Lucanidae nested within the Scarabaeidae. In the present version, there are no trees with Lucanidae nested within the Scarabaeidae. The associated sentence has been deleted.

Q4: Ultimately, while I think there are elements of this paper that are novel- the vast majority of this is not. The novelty in this paper revolves around the new genomes, including their descriptions of structure, unfortunately the particular species sequenced do not add novel insights in to poorly resolved relationships in Scarabaeidae. Large components of this paper are just a reanalysis of previously published data from other papers. The results and discussion to not add any new insights in to relationships within the family or subfamilies. For example, the entire section on tribal

relationships and nesting behaviors is unnecessary as it is a synthesis of other work that has been discussed in detail in other, sometimes uncited, papers.

Response: The paper of Breeshoten et al. (2016) has been cited in this version (Page 15 Lines 390-391, and Page 16 Lines 397-399). We added some discussions on the comparisons between our results and theirs. With regard to the comments from another reviewer, we added the tribe labels in Figures 4-5 and retained this section in the text.

Q5: Furthermore, I disagree with the statement on line 364 that it's sufficiently sampled (beyond the ingroup of Breeshoten's work) to address internal relationships, as a number of tribes are not includes, are represented by a single taxon or multiple species in the same genus. I suspect the authors are not familiar with much of the scarab literature because other unanswered questions / unconfirmed relationships in the Scarabaeidae, such as the putative paraphyly of Rutelinae, were not discussed.

Response: In this version, we compared the taxon sampling of Rutelinae with the two prior studies of Ahrens 2014 and Gunter 2016. For the monophyly of Rutelinae, we have added new discussions in Page 14 Lines 350-366.

Q6: I would have liked to see a summary of taxon sampling within the text. The figures were neat and informative. Please note that some taxonomic names within the phylogeny are out of date, for example *Digonthophaagus bonasus*.

Response: The species name *Onthophagus bonasus* is from NCBI database (KU739459) https://www.ncbi.nlm.nih.gov/nuccore/KU739459.1/.

In this version, we have corrected *Onthophagus bonasus* to *Digitonthophagus bonasus* throughout the trees.

Reviewer #2:

Q1: 1). Coprophagy. Aphodinae and a significant minority of Scarabaeinae aren't strict dung feeders.

They are generalist saprovores, consuming dead/rotting plant material of which dung is a specialised dietary type (this is reflected in the fact that few scarabaeine dung beetle consume dung from non-herbivorous vertebrates). These dietary distinctions and their evolution are discussed at length in Gunter et al. 2016 PLoS One 11 e0153570. It is also worth noting that these distinctions exclusively concern adult diet - almost all Scarabs are saprovores as larvae, specialisation between phyto, sapro and coprophagy is only found in adults.

Response: Thanks for this comment. The corresponding statements have been revised and the paper of Gunter et al. 2016 has been cited in this version. Please see details in Page 3 Lines 42-44, 52-55.

Q2: 2) Literature comparisons. The taxon sampling used here, while extensive from the mt genome point of view, is restricted relative to the available molecular phylogenies of the family. For example, just 3sp of Aphedinae, 6 Rutelinae (and just 3 genera) etc. The comparisons to prior literature thus need to be more refined that direct equivalence of subfamily or tribes between the present study and past ones.

It is necessary to examine if the representatives within each higher taxon are comparable studies, or if the ones included here represent only particular clades included in other studies.

For example Rutelinae is paraphyletic in both Gunter 2016 and Ahrens 2014, forming 3 clades and Dynastinae within the Rutelinae. The relationships found in the present study could be completely accurate if only species from 1 of those three clades were included i.e. both subfamilies monophletic, it would just be misleading as Rutelinae is undersampled to test those prior relationships. Similar issues with the Scarabaeinae.

Response: In this version, we compared the taxon sampling of Rutelinae with the two prior studies of Ahrens 2014 and Gunter 2016. For the monophyly of Rutelinae, we have added new discussions in Page 14 Lines 350-366.

Q3: I would like to see a more detailed examination of how well sampled clades are rather than just statements as to monophyly and relationship with other clades.

Additionally, as devote substantial discussion space to tribal relationships in Scarabaeinae, tribe labels should be labelled on your figure for ease of reading. This section could also benefit from

more extensive use of recent literature on behavioural evolution as noted above for Coprophagy.

Response: We compared the taxon sampling of the major clade within Scarabaeidae included in this study to the prior studies, particularly the studies of Breeshoten et al. (2016), Ahrens et al. (2014) and Gunter et al. (2016). We added the discussions on the tribe relationships within Scarabaeinae (Page 15 Lines 390-391, and Page 16 Lines 397-399) and the monophyly of Rutelinae (Page 14 Lines 350-366). In addition, we added the tribe labels in Figures 4-5.
