# Supplementary material for: Mitochondrial phylogenomics reveals deep relationships of scarab beetles (Coleoptera, Scarabaeidae)
Source: PLoS One. 2022 Dec 13;17(12):e0278820. doi: 10.1371/journal.pone.0278820 (PMC9746968; doi:10.1371/journal.pone.0278820)
Supplement: Attachment — Submitted filename: Responses to comments_20220921.docx [file pone.0278820.s014.docx]

# Responses to comments

Dear Reviewers,

Thank you for your very careful review of our paper, and for the comments, corrections and suggestions that ensued. A major revision of the paper has been carried out to take all of them into account. And in the process, we believe the paper has been significantly improved.

In the present “Responses to comments”, we sequentially address all of the points raised in each of the comments made by different reviewers.

## Reviewer #1:

**Q1**: Breeschoten et al. 2016 for which much of the included sequences were published in,

**Response:** This reference has been cited in Table S1.

In the old version, we find the references associated with the mitogenome sequences from GenBank. Although much of the included sequences are from the Breeschoten et al. 2016, the references in GenBank are not updated where the state of “JOURNAL” is still “Unpublished”. According to your comment, we read the paper of Breeschoten et al. 2016 and checked the mitogenome sequences included in it. Indeed, much of the included sequences come from this paper.

Thank you for this comment.

**Q2**: phylogenies that address the monophyly of the Scarabaeidae including McKenna et al. 2014 and Gunter et al. 2016 especially the most recent papers with dense gene or taxon sampling- are relatively well resolved and provide a broader snap-shot of the deep level relationship that this paper attempts to address.

**Response:** After careful inspection, we found that McKenna et al. 2014 you mentioned might be the one of McKenna et al. 2015 (please see details in the following).

McKenna DD, Farrell BD, Caterino MS, Farnum CW, Hawks DC, Maddison DR, et al. Phylogeny and evolution of Staphyliniformia and Scarabaeiformia: forest litter as a stepping-stone for diversification of non-phytophagous beetles. Syst. Entomol. 2015; 40: 35–60. doi: 10.1111/syen.12093

This paper included the content of addressing the monophyly of the Scarabaeidae. This reference has been cited in this version. Page 12 Line 303, and Page 15 Lines 395-396.

In addition, we also read carefully the paper of Gunter et al. 2016 and cited it in this version. Page 3 Lines 52, 55 and Page 14 Lines 356-360.

**Q3**: It is extremely surprising to me that the Lucanidae rendered Scarabaeidae para-phyletic in some analysis. It is generally considered one of the earliest branching members of the superfamily as supported by molecular result and the deep fossil record. This surprising result may indicate that the mt-genome phylogeny is either insufficiently sampled at a taxon-level or is inappropriate for resolving relationships. When analyzed by amino acids and removing the 3rd codon results were more congruent with past analyses.

**Response:** I am sorry that this is a mistake in writing. In the preliminary analysis on the datasets with the different taxon sampling, some resulting trees might please Lucanidae nested within the Scarabaeidae. In the present version, there are no trees with Lucanidae nested within the Scarabaeidae. The associated sentence has been deleted.

**Q4**: Ultimately, while I think there are elements of this paper that are novel- the vast majority of this is not. The novelty in this paper revolves around the new genomes, including their descriptions of structure, unfortunately the particular species sequenced do not add novel insights in to poorly resolved relationships in Scarabaeidae. Large components of this paper are just a reanalysis of previously published data from other papers. The results and discussion to not add any new insights in to relationships within the family or subfamilies. For example, the entire section on tribal relationships and nesting behaviors is unnecessary as it is a synthesis of other work that has been discussed in detail in other, sometimes uncited, papers.

**Response:** The paper of Breeshoten et al. (2016) has been cited in this version (Page 15 Lines 390-391, and Page 16 Lines 397-399). We added some discussions on the comparisons between our results and theirs. With regard to the comments from another reviewer, we added the tribe labels in Figures 4-5 and retained this section in the text.

**Q5**: Furthermore, I disagree with the statement on line 364 that it’s sufficiently sampled (beyond the ingroup of Breeshoten’s work) to address internal relationships, as a number of tribes are not includes, are represented by a single taxon or multiple species in the same genus. I suspect the authors are not familiar with much of the scarab literature because other unanswered questions / unconfirmed relationships in the Scarabaeidae, such as the putative paraphyly of Rutelinae, were not discussed.

**Response:** In this version, we compared the taxon sampling of Rutelinae with the two prior studies of Ahrens 2014 and Gunter 2016. For the monophyly of Rutelinae, we have added new discussions in Page 14 Lines 350-366.

**Q6**: I would have liked to see a summary of taxon sampling within the text. The figures were neat and informative. Please note that some taxonomic names within the phylogeny are out of date, for example *Digonthophaagus bonasus*.

**Response:** The species name *Onthophagus bonasus* is from NCBI database (KU739459) https://www.ncbi.nlm.nih.gov/nuccore/KU739459.1/.

In this version, we have corrected *Onthophagus bonasus* to *Digitonthophagus bonasus* throughout the trees.

## Reviewer #2:

**Q1:** 1). Coprophagy. Aphodinae and a significant minority of Scarabaeinae aren't strict dung feeders. They are generalist saprovores, consuming dead/rotting plant material of which dung is a specialised dietary type (this is reflected in the fact that few scarabaeine dung beetle consume dung from non-herbivorous vertebrates). These dietary distinctions and their evolution are discussed at length in Gunter et al. 2016 PLoS One 11 e0153570. It is also worth noting that these distinctions exclusively concern adult diet - almost all Scarabs are saprovores as larvae, specialisation between phyto, sapro and coprophagy is only found in adults.

**Response:** Thanks for this comment. The corresponding statements have been revised and the paper of Gunter et al. 2016 has been cited in this version. Please see details in Page 3 Lines 42-44, 52-55.

**Q2:** 2) Literature comparisons. The taxon sampling used here, while extensive from the mt genome point of view, is restricted relative to the available molecular phylogenies of the family. For example, just 3sp of Aphedinae, 6 Rutelinae (and just 3 genera) etc. The comparisons to prior literature thus need to be more refined that direct equivalence of subfamily or tribes between the present study and past ones.

It is necessary to examine if the representatives within each higher taxon are comparable studies, or if the ones included here represent only particular clades included in other studies.

For example Rutelinae is paraphyletic in both Gunter 2016 and Ahrens 2014, forming 3 clades and Dynastinae within the Rutelinae. The relationships found in the present study could be completely accurate if only species from 1 of those three clades were included i.e. both subfamilies monophletic, it would just be misleading as Rutelinae is undersampled to test those prior relationships. Similar issues with the Scarabaeinae.

**Response:** In this version, we compared the taxon sampling of Rutelinae with the two prior studies of Ahrens 2014 and Gunter 2016. For the monophyly of Rutelinae, we have added new discussions in Page 14 Lines 350-366.

**Q3:** I would like to see a more detailed examination of how well sampled clades are rather than just statements as to monophyly and relationship with other clades.

Additionally, as devote substantial discussion space to tribal relationships in Scarabaeinae, tribe labels should be labelled on your figure for ease of reading. This section could also benefit from more extensive use of recent literature on behavioural evolution as noted above for Coprophagy.

**Response:** We compared the taxon sampling of the major clade within Scarabaeidae included in this study to the prior studies, particularly the studies of Breeshoten et al. (2016), Ahrens et al. (2014) and Gunter et al. (2016). We added the discussions on the tribe relationships within Scarabaeinae (Page 15 Lines 390-391, and Page 16 Lines 397-399) and the monophyly of Rutelinae (Page 14 Lines 350-366). In addition, we added the tribe labels in Figures 4-5.
