# Supplementary material for: Mitochondrial phylogenomics reveals deep relationships of scarab beetles (Coleoptera, Scarabaeidae)
Source: PLoS One. 2022 Dec 13;17(12):e0278820. doi: 10.1371/journal.pone.0278820 (PMC9746968; doi:10.1371/journal.pone.0278820)
Supplement: Attachment — Submitted filename: Response to Reviewers.docx [file pone.0278820.s016.docx]

# Response to reviewers

Dear Reviewers,

Thank you for your very careful review of our paper, and for the comments, corrections and suggestions that ensued. A minor revision of the paper has been carried out to take all of them into account. And in the process, we believe the paper has been significantly improved.

In the present “Responses to comments”, we sequentially address all of the points raised in each of the comments made by different reviewers.

## Reviewer #1:

**Q1.** It would be very helpful to identify the *Amphimallon* species. Best practices would be to deposit both specimens in a museum as a voucher that is available for subsequent verification.

**Response:** Because the insect of this species is large, only leg was used for DNA extraction. The remaining specimen with a relatively complete insect body has been deposited in Entomological Museum of Henan Agricultural University (voucher number: *Amphimallon* sp., EMHAU-2022-Zz210705). The corresponding statements have been added in this version (Page 6 Lines 136-140).

**Q2.** The discussion of monophyly of Aphodiinae could be improved as many papers recover it as a paraphyletic grade and not monophyletic.

**Response:** The discussions and references on the monophyly of Aphodiinae have been added in this version (Page 14 Lines 340-350).

## Reviewer #3:

**Q1.** The conclusion part seems to be incomplete after all the molecular work, as the final inferences does not include the significance and applicability of the work in the present scenario. Also, some evolutionary aspects in form of final lineage and its relatedness with important model organisms should be mentioned.

**Response:** The relevant conclusions have been added in the section of conclusion (Page 17 Lines 452-453, and Page 18 Lines 454-456, 462-464).

**Q2.** Futuristic approach also should be addressed compliantly in relation with the present research work as designed by the Authors.

**Response:** The associated statements have been added in Page 14 Lines 348-350 and in the section of conclusion (Page 18 Lines 454-456).
